# Pathogen diversity and antimicrobial resistance transmission of *Salmonella enterica* serovars Typhi and Paratyphi A in Bangladesh, Nepal, and Malawi: a genomic epidemiological study

**DOI:** 10.1016/S2666-5247(24)00047-8

**Published:** 2024-08

**Authors:** Zoe A Dyson, Philip M Ashton, Farhana Khanam, Angeziwa Chunga Chirambo, Mila Shakya, James E Meiring, Susan Tonks, Abhilasha Karkey, Chisomo Msefula, John D Clemens, Sarah J Dunstan, Stephen Baker, Gordon Dougan, Virginia E Pitzer, Buddha Basnyat, Firdausi Qadri, Robert S Heyderman, Melita A Gordon, Andrew J Pollard, Kathryn E Holt, STRATAA Study Group, Happy C Banda, Prasanta K Biswas, Md A I Bhuiyan, Christoph Blohmke, Thomas C Darton, Christiane Dolecek, Sabina Dongol, Yama F Mujadidi, Jennifer Hill, Nhu T Hoang, Tikhala M Jere, Maurice Mbewe, Harrison Msuku, Tran V T Nga, Rose Nkhata, Sadia IA Rahman, Nazia Rahman, Neil J Saad, Trinh V Tan, Deus Thindwa, Merryn Voysey, Richard Wachepa

**Affiliations:** r Malawi-Liverpool Wellcome Programme, Blantyre, Malawi; s International Centre for Diarrhoeal Disease Research, Dhaka, Bangladesh; t Oxford Vaccine Group, Department of Paediatrics, University of Oxford, and the NIHR Oxford Biomedical Research Centre, Oxford, United Kingdom; u Nuffield Department of Medicine, Centre for Tropical Medicine and Global Health, University of Oxford, Oxford, UK; v Oxford University Clinical Research Unit, Patan Academy of Health Sciences, Kathmandu, Nepal; w The Hospital for Tropical Diseases, Wellcome Trust Major Overseas Programme, Oxford University Clinical Research Unit, Ho Chi Minh City, Vietnam; x Department of Epidemiology of Microbial Diseases and the Public Health Modeling Unit, Yale School of Public Health, Yale University, New Haven, CT, USA; a Department of Infection Biology, Faculty of Infectious and Tropical Diseases, London School of Hygiene & Tropical Medicine, London, UK; b Department of Infectious Diseases, Central Clinical School, Monash University, Melbourne, VIC, Australia; c Wellcome Sanger Institute, Wellcome Genome Campus, Hinxton, UK; d Malawi–Liverpool–Wellcome Programme, Blantyre, Malawi; e Institute of Infection, Veterinary & Ecological Sciences, University of Liverpool, Liverpool, UK; f International Centre for Diarrhoeal Disease Research, Dhaka, Bangladesh; g Kamuzu University of Health Sciences, Blantyre, Malawi; h Oxford University Clinical Research Unit, Patan Academy of Health Sciences, Kathmandu, Nepal; i Oxford Vaccine Group, Department of Paediatrics, University of Oxford, and the NIHR Oxford Biomedical Research Centre, Oxford, UK; j Department of Infection, Immunity and Cardiovascular Disease, University of Sheffield, Sheffield, UK; k Centre for Tropical Medicine and Global Health, Medical Sciences Division, Nuffield Department of Medicine, University of Oxford, Oxford, UK; l International Vaccine Institute, Seoul, South Korea; m Department of Infectious Diseases, University of Melbourne at the Peter Doherty Institute for Infection and Immunity, Melbourne, VIC, Australia; n Department of Medicine, Cambridge Institute of Therapeutic Immunology and Infectious Diseases, University of Cambridge, Cambridge, UK; o Department of Epidemiology of Microbial Diseases and the Public Health Modeling Unit, Yale School of Public Health, Yale University, New Haven, CT, USA; p NIHR Global Health Research Unit on Mucosal Pathogens, Division of Infection and Immunity, University College London, London, UK; q Department of Clinical Sciences, Liverpool School of Tropical Medicine, Liverpool, UK

## Abstract

**Background:**

Enteric fever is a serious public health concern. The causative agents, *Salmonella enterica* serovars Typhi and Paratyphi A, frequently have antimicrobial resistance (AMR), leading to limited treatment options and poorer clinical outcomes. We investigated the genomic epidemiology, resistance mechanisms, and transmission dynamics of these pathogens at three urban sites in Africa and Asia.

**Methods:**

*S* Typhi and *S* Paratyphi A bacteria isolated from blood cultures of febrile children and adults at study sites in Dhaka (Bangladesh), Kathmandu (Nepal), and Blantyre (Malawi) during STRATAA surveillance were sequenced. Isolates were charactered in terms of their serotypes, genotypes (according to GenoTyphi and Paratype), molecular determinants of AMR, and population structure. We used phylogenomic analyses incorporating globally representative genomic data from previously published surveillance studies and ancestral state reconstruction to differentiate locally circulating from imported pathogen AMR variants. Clusters of sequences without any single-nucleotide variants in their core genome were identified and used to explore spatiotemporal patterns and transmission dynamics.

**Findings:**

We sequenced 731 genomes from isolates obtained during surveillance across the three sites between Oct 1, 2016, and Aug 31, 2019 (24 months in Dhaka and Kathmandu and 34 months in Blantyre). *S* Paratyphi A was present in Dhaka and Kathmandu but not Blantyre. *S* Typhi genotype 4.3.1 (H58) was common in all sites, but with different dominant variants (4.3.1.1.EA1 in Blantyre, 4.3.1.1 in Dhaka, and 4.3.1.2 in Kathmandu). Multidrug resistance (ie, resistance to chloramphenicol, co-trimoxazole, and ampicillin) was common in Blantyre (138 [98%] of 141 cases) and Dhaka (143 [32%] of 452), but absent from Kathmandu. Quinolone-resistance mutations were common in Dhaka (451 [>99%] of 452) and Kathmandu (123 [89%] of 138), but not in Blantyre (three [2%] of 141). Azithromycin-resistance mutations in *acrB* were rare, appearing only in Dhaka (five [1%] of 452). Phylogenetic analyses showed that most cases derived from pre-existing, locally established pathogen variants; 702 (98%) of 713 drug-resistant infections resulted from local circulation of AMR variants, not imported variants or recent de novo emergence; and pathogen variants circulated across age groups. 479 (66%) of 731 cases clustered with others that were indistinguishable by point mutations; individual clusters included multiple age groups and persisted for up to 2·3 years, and AMR determinants were invariant within clusters.

**Interpretation:**

Enteric fever was associated with locally established pathogen variants that circulate across age groups. AMR infections resulted from local transmission of resistant strains. These results form a baseline against which to monitor the impacts of control measures.

**Funding:**

Wellcome Trust, Bill & Melinda Gates Foundation, EU Horizon 2020, and UK National Institute for Health and Care Research.

## Introduction

The global burden of enteric fever is estimated at 14·3 million cases annually,^1^ concentrated in south Asia and sub-Saharan Africa. The causative agents are *Salmonella enterica* serovars Typhi and Paratyphi A. Mortality and complications occur at higher rates in the absence of effective antimicrobial therapy. Antimicrobial resistance (AMR) is common, but the specific drugs and mechanisms vary. In most regions, resistance to the former first-line drugs chloramphenicol, co-trimoxazole, and ampicillin (the combination of which is defined as multidrug resistance [MDR]) has declined, and fluoroquinolone non-susceptibility (ciprofloxacin minimum inhibitory concentration ≥0·06 mg/L)^2^^,^^3^ has increased. Resistance to cefixime and azithromycin is emerging, mainly in south Asia,^4^, ^5^, ^6^, ^7^ and *S* Typhi with extensive drug resistance (defined as MDR plus resistance to ciprofloxacin and third-generation cephalosporins) is common in Pakistan.^4^ Regional transfer and local clonal expansion of AMR variants have been documented,^7^, ^8^, ^9^, ^10^ but the extent to which AMR disease burden is attributable to the transmission of endemic variants, as opposed to the importation of exogenous variants or de novo emergence of AMR in the local population, has not been specifically quantified.

**Research in context**

**Evidence before this study**

Current knowledge of the enteric fever pathogen populations in Dhaka, Kathmandu, and Blantyre comes from retrospective analyses of isolates captured from routine diagnostics or treatment trials. Due to these study designs, most focus on either adult or paediatric cohorts, which complicates assessment of pathogen variant transmission across age groups. Many studies report prevalence of antimicrobial resistance (AMR) and associated mechanisms amongst enteric fever cases. Genomic studies at these sites and elsewhere have identified the spread of AMR clones, and a recent genomic study quantified the intercontinental and intracontinental spread of resistant *S* Typhi between countries. However, a PubMed search for articles published between Jan 1, 2001, and Nov 1, 2022, for “(typhoid OR (enteric fever)) AND (genom∗)” identified no studies quantifying the relative proportion of resistant infections that is attributable to local transmission of resistant variants versus imported strains or de novo emergence of AMR.

**Added value of this study**

We estimated that the vast majority (98%) of drug-resistant enteric fever cases identified in our study resulted from local circulation of resistant variants. Furthermore, we showed genetically indistinguishable pathogen variants (either resistant or susceptible) persisting for up to 2·3 years and causing infections across all age groups (<5 years, 5 to <15 years, and ≥15 years).

**Implications of all the available evidence**

While intercountry transfer of resistant enteric fever pathogens does occur and is concerning, the burden of drug-resistant enteric fever at the study sites is currently caused mainly by transmission of locally established variants, and transmits across age groups. These data confirm assumptions made in models of vaccine impact regarding heterogeneity of pathogen variants and AMR across age groups, and support that childhood immunisation programmes can be expected to reduce the overall burden of resistant infections in endemic settings.

Vaccines against *S* Typhi have been used for travellers for decades, but mass immunisation has not been applied in most endemic areas. Recently licensed Gavi-supported typhoid conjugate vaccines (TCVs) offer new opportunities to reduce disease burden.^11^ Trials have shown these vaccines to be safe and immunogenic in children (from age 9 months), with more than 80% efficacy.^12^, ^13^, ^14^, ^15^ In Pakistan and Zimbabwe, TCV immunisation campaigns have been deployed to control extensively drug-resistant and ciprofloxacin-resistant *S* Typhi outbreaks.^16^^,^^17^ These successful campaigns were followed by the introduction of national immunisation programmes,^18^^,^^19^ which are now being considered by several other countries.

It is important to monitor the impact of vaccine programmes on pathogen populations. TCVs do not cross-protect against *S* Paratyphi A, which could expand to fill the niche. TCVs are effective against some AMR *S* Typhi variants,^18^^,^^19^ but it is unknown whether they will be equally effective against all variants or promote the emergence of vaccine-escape mutants or AMR variants. Baseline data are therefore essential; whole-genome sequencing is now the standard as it provides high-resolution data on lineage diversity, resistance mechanisms, and transmission dynamics. We previously assessed the burden of enteric fever in three urban sites (Blantyre, Malawi; Kathmandu, Nepal; and Dhaka, Bangladesh) as part of the Strategic Typhoid Alliance across Africa and Asia (STRATAA).^20^ Current knowledge of the enteric fever pathogen populations at STRATAA sites comes from retrospective analyses of isolates captured from routine diagnostics^3^ or treatment trials^10^ conducted using different protocols, and usually separated into adult or paediatric studies.^3^^,^^10^ These data indicate that *S* Typhi 4.3.1 genotypes (H58) have been dominant across south Asia and eastern and southern Africa, including STRATAA sites, for many years.^2^^,^^3^^,^^8^, ^9^, ^10^ In Malawi, *S* Typhi epidemics have been documented since the 1990s and are now associated almost entirely with the MDR genotype 4.3.1, which has been clonally expanding since its arrival in 2009, and the disease is now considered endemic.^8^^,^^9^ By contrast, in Bangladesh and Nepal, *S* Typhi and *S* Paratyphi A have been hyperendemic for decades, and probably centuries, reflected in a diversity of cocirculating pathogen genotypes, although the populations have been dominated by *S* Typhi genotype 4.3.1 for the past two decades.^2^^,^^3^^,^^21^

In this study, we used whole-genome sequencing (WGS) to investigate the pathogen populations underlying enteric fever at STRATAA sites, collected prospectively in defined catchment areas using the same protocol, providing baseline data ahead of vaccine trials^12^, ^13^, ^14^, ^15^ and planned immunisation programmes.^19^ We characterised pathogen populations and AMR determinants, investigated transmission patterns across age groups, and quantified the proportion of AMR cases attributable to local transmission of endemic AMR variants.

## Methods

### Study design and participants

In this genomic epidemiological study, the three study sites in Blantyre, Dhaka, and Kathmandu were selected from locations across Africa and Asia on the basis of their known high rates of enteric fever and capacity to deliver a large-scale and logistically complex study. During the initial census period and during repeat census of the population, participants enrolled into the census by the study fieldwork teams were informed to attend the study health clinics when febrile.^22^ Those presenting with fever for at least 48 h or temperature of at least 38·0°C were approached for recruitment.^22^ For analyses presented here, the inclusion criteria were blood culture-confirmed cases of enteric fever due to *S* Typhi or *S* Paratyphi A derived from separate individuals resident within the census area, collected within the surveillance periods. Clinical data from passive surveillance were collected using a combination of electronic and paper forms, with paper forms transcribed into electronic databases using OpenClinica. Global Positioning System (GPS) coordinates for participants’ residences were collected using forms developed with Open Data Kit and stored in MySQL databases. The full STRATAA protocol^22^ and burden data^20^ are published elsewhere, and additional details of passive surveillance are provided in appendix 1 (p 2).

Research ethics committee approval for a joint study protocol^22^ across all three surveillance sites was obtained within each country and from the Oxford Tropical Research Ethics Committee (University of Oxford, Oxford, UK).^20^ Written informed consent was obtain from participants or their guardians.

### Procedures

Isolates cultured from blood samples from febrile individuals recruited into STRATAA passive surveillance studies were stored locally until the end of the recruitment period. Subsequently, isolates were cultured overnight and genomic DNA extracted using Wizard Genomic DNA Extraction Kits following the manufacturer’s recommendations (Promega, Madison, WI, USA). DNA was shipped to the Wellcome Sanger Institute and subjected to indexed WGS on an Illumina HiSeq 2500 to generate paired-end reads (100 bp).^8^ Single-nucleotide variants (SNVs) were identified by mapping sequence reads to the *S* Typhi CT18 (accession number AL513382) and *S* Paratyphi A AKU_12601 (accession number FM200053) reference genomes using RedDog (V1beta.11). Genotypes were assigned using GenoTyphi (v1.9.1)^23^ and Paratype (v1_beta2). Recombination-filtered maximum-likelihood phylogenies, including 3128 *S* Typhi and 258 *S* Paratyphi A genome sequences from global collections (appendix 2) to support differentiation of imported versus locally transmitted AMR, were inferred using Gubbins (v2.4.1) and RAxML (v8.2.8). AMR genes and plasmids were detected using SRST2 (v0.2.0). To determine whether molecular determinants of AMR were transmitted or inherited, we did maximum-parsimony ancestral state reconstruction of AMR determinants and country using the R package phangorn (v2.5.5). Transmission dynamics were explored by comparing the spatial, temporal, and epidemiological features of clusters of genomes between which zero core-genome SNVs were detected (zero-SNV clusters, identified based on pairwise SNV distances extracted from the SNV alignment using disty [v0.1.0]). GPS coordinates were collected by fieldworkers from the homes of participants using either study tablets or GPS machines. If a GPS signal could not be obtained at a particular location due to density, then the closest location where a signal could be obtained was recorded. Spatial pairwise distances were calculated using the geopy.distance.distance function from the geopy Python package (v2.3.0). Antimicrobial susceptibility testing was done with use of disc-diffusion (appendix 1 p 2).^20^

### Statistical analysis

Statistical tests were conducted in R (v4.1.2) unless otherwise stated, using two-sided tests and with p<0·05 considered significant. Positive and negative predictive values were calculated for comparisons between molecular determinants of AMR and associated phenotypes using the epi.tests() function in the R package epiR (v2.0.62). Pathogen serotype and genotype distributions were assessed by site, age group and sex (per site), and longitudinally (per site). Age was treated as a categorical variable (grouped into <5 years, ≥5 and <15 years, ≥15 years) and sex as a binary variable (male or female). Genotype diversity (per serotype) was assessed by site and by age group using Simpson’s index with 95% CIs, calculated using the R packages vegan (v2.5.6) and iNEXT (v3.0.0), as described in appendix 1 (p 3). Severe disease (defined as symptom duration >10 days or requirement for hospitalisation, based on clinical review of case report forms) was assessed by age group and sex (per site). For *S* Typhi cases, the association between severe disease (binary outcome) and predictors (age group, sex, H58 genotype [binary], and MDR [binary]) were assessed using penalised-likelihood logistic regression models fit using the logistf package (v1.24.1), with odds ratio (OR) and 95% CI as a measure of association. The same approach was used to test for association between MDR prevalence (binary outcome) and predictors (age group and sex). Clustering of age groups on the phylogenies was assessed using the *K* statistic, calculated using the function multiPhylosignal() in the R package picante (v1.8.2). Transmission dynamics were assessed by comparing distributions of pairwise spatial and temporal distances between cases in zero-SNV clusters versus unclustered cases using the Kolmogorov–Smirnov two-sample test, implemented in the scipy (v1.7.1) package of Python (scipy.stats.ks_2samp). Associations between an individual genotype (binary) or age groups and membership of a zero-SNV cluster (*vs* unclustered cases, binary variable) were assessed using χ^2^ tests.

### Role of the funding source

The funders of the study had no role in study design, data collection, data analysis, data interpretation, or writing of the report.

## Results

We recruited 776 febrile individuals in STRATAA catchment areas during passive surveillance: 454 in Dhaka, 164 in Kathmandu, and 158 in Blantyre. The surveillance periods were from Jan 1, 2017, to Dec 31, 2018, in Dhaka and Kathmandu, and from Oct 1, 2016, to Sept 30, 2018, in Blantyre. An additional 10 months of surveillance data (to Aug 31, 2019) were available for Blantyre (43 of the total samples from this catchment), and were used to better understand these populations, but these data were not included in diversity analyses to prevent bias (table 1). Isolates from 45 participants could not be sequenced. We sequenced 731 unique typhoidal *Salmonella* blood-culture isolates^20^^,^^22^ (table 1; appendix 2). Serotype and genotype distributions are shown in table 1. Genomic analysis confirmed^20^ that *S* Paratyphi A was present in the south Asian sites (95 [21%] of 452 in Dhaka and 14 [10%] of 138 in Kathmandu), but not in Blantyre. Across all three sites, *S* Typhi 4.3.1 (H58) genotypes were most frequently observed, representing between 42% (191 of 452) and 98% (138 of 141) of sequenced isolates (table 1), consistent with previous studies.^2^^,^^3^^,^^8^, ^9^, ^10^ However, different H58 subtypes were present at each of the sites, which also differed markedly in terms of the diversity of pathogen variants causing enteric fever (table 1; appendix 1 p 6). Enteric fever cases in Blantyre were mostly caused by *S* Typhi 4.3.1.1.EA1 (138 [98%] of 141), which displayed low genotype diversity (Simpson’s index 0·05 [95% CI 0·05–0·11]). Enteric fever cases in Kathmandu were more diverse (Simpson’s index 0·67 [0·67–0·73]), with 41 (30%) of 138 cases caused by the non-H58 *S* Typhi genotype 3.3.2 and 67 (49%) caused by *S* Typhi genotype 4.3.1.2 (table 1; appendix 1 p 6). Dhaka was the most diverse setting (Simpson’s index 0·80 [0·80–0·83]), with two common lineages of H58 *S* Typhi, genotypes 4.3.1.1 (177 [39%] of 452) and 4.3.1.3.Bdq (12 [3%] of 452), in addition to four non-H58 *S* Typhi genotypes with at least 25 cases each (2.0.1, 2.1.7, 2.3.3, 3.2.2) and two common *S* Paratyphi A genotypes with at least 20 cases (2.3.3 and 2.4.4; table 1; appendix 1 p 6).

**Table 1:** Summary of enteric fever cases and sequenced pathogens

	Dhaka	Kathmandu	Blantyre∗	
			24 months	34 months
**Recruitment**				
Surveillance period	Jan 1, 2017–Dec 31, 2018 (24 months)	Jan 1, 2017–Dec 31, 2018 (24 months)	Oct 1, 2016–Sept 30, 2018	Oct 1, 2016–Aug 31, 2019
Unique participants recruited	454	164	115	158
Unique participants with available sequencing data	452/454 (>99%)	138/164 (84%)	83/115 (72%)	141/158 (89%)
**Characteristics of participants with sequencing data**				
Sex				
Male	240/452 (53%)	82/138 (59%)	33/83 (40%)	64/141 (45%)
Female	212/452 (47%)	56/138 (41%)	50/83 (60%)	77/141 (55%)
Age, years				
<5 years	105/452 (23%)	10/138 (7%)	17/83 (20%)	27/141 (19%)
≥5 and <15 years	208/452 (46%)	60/138 (43%)	48/83 (58%)	72/141 (51%)
≥15 years	139/452 (31%)	68/138 (49%)	18/83 (22%)	42/141 (30%)
Simpson’s diversity (95% CI)	0·80 (0·80–0·83)	0·67 (0·67–0·73)	0·05 (0·05–0·11)	··
***S* Typhi**				
Total sequences	357/452 (79%)	124/138 (90%)	83/83 (100%)	141/141 (100%)
4.3.1 (H58)	191/357 (54%)	80/124 (65%)	81/83 (98%)	138/141 (98%)
4.3.1.1	177/357 (50%)	3/124 (2%)	81/83 (98%)†	138/141 (98%)†
4.3.1.2	2/357 (1%)	67/124 (54%)	0/83 (0%)	0/141 (0%)
2.3.3	64/357 (18%)	0/124 (0%)	0/83 (0%)	0/141 (0%)
3.3.2	17/357 (5%)	41/124 (33%)	0/83 (0%)	0/141 (0%)
Simpson’s diversity (95% CI)	0·70 (0·70–0·75)	0·59 (0·59–0·66)	0·05 (0·05–0·11)	··
***S* Paratyphi A**				
Total sequences	95/452 (21%)	14/138 (10%)	0/83 (0%)	0/141 (0%)
2.3.2	11/95 (12%)	4/14 (29%)	··	··
2.3.3	23/95 (24%)	0/14 (0%)	··	··
2.4.1	3/95 (3%)	3/14 (21%)	··	··
2.4.3	0/95 (0%)	6/14 (43%)	··	··
2.4.4	56/95 (59%)	0/14 (0%)	··	··
Simpson’s diversity (95% CI)	0·58 (0·58–0·67)	0·68 (0·68–0·88)	··	··

Data are n/N (%). For each *Salmonella enterica* serovar (*S* Typhi, *S* Paratyphi A), the table shows total sequenced cases; number and frequency of common genotypes (those accounting for ≥10% total sequenced cases in at least one site); and Simpson’s diversity index (calculated from genotype counts).

∗ Blantyre had an extended surveillance period; data from the full period (34 months) is included in the main STRATAA dataset used for phylogenetic and statistical analyses (n=622 *S* Typhi and n=109 *S* Paratyphi A); however, the first 24-month period was used to calculate Simpson’s diversity so as to be comparable across sites.

† Sublineage 4.3.1.1.EA1.

**Figure 1:**
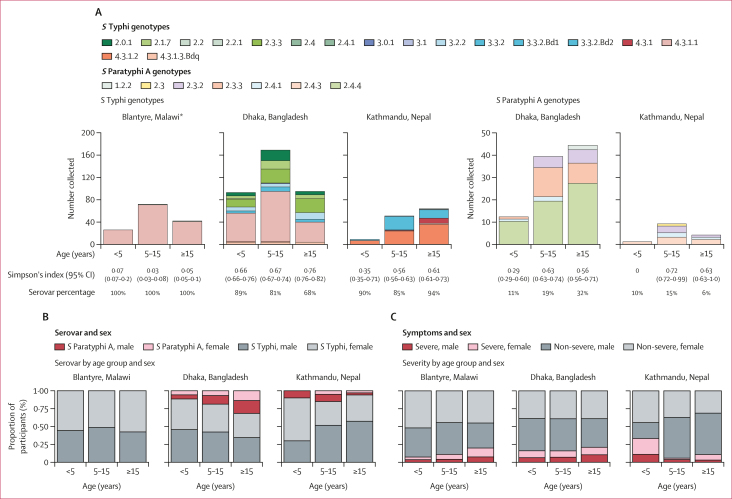
Distribution of pathogen variants and disease severity by age group (A) Barplots show frequency distributions of pathogen genotypes among age groups, stratified by serovar and location, for the main STRATAA dataset (n=622 *Salmonella enterica* serovar Typhi and n=109 *Salmonella enterica* serovar Paratyphi A; table 1). Simpson’s diversity index is shown under each bar (calculated from genotype counts, excluding isolates from the extended surveillance period in Blantyre to ensure comparability between locations), along with the frequencies of each serovar (*S* Typhi or *S* Paratyphi A) as a percentage of all sequenced isolates from the given location and age group. (B) Breakdown of patient sex and pathogen serovar, within each age group at each site, for the main STRATAA dataset. (C) Breakdown of patient sex and disease severity (severe was defined as a symptom duration >10 days or requirement for hospitalisation), within each age group at each site, for the main STRATAA dataset. ∗4.3.1.1 in Blantyre is sublineage 4.3.1.1.EA1.

We considered three age groups that reflect broadly different contact networks: pre-school age children (<5 years and interacting mainly with other household members), school-age children (5–15 years, interacting with household members and other school-age children), and working age (≥15 years, interacting with household members and the wider community). In all settings, all age groups were infected with a diverse range of *S* Typhi and *S* Paratyphi A genotypes (figure 1A). Similarly, at the sub-genotype level, the age groups were intermingled in the phylogenetic trees (appendix 1 pp 11–13), with no evidence of clustering by age group (*K*=7·16 × 10^–6^ to 2·24 × 10^–5^; *K*<1 indicates no signal). Within each site, local prevalence of *S* Paratyphi A and H58 *S* Typhi, were similar across age groups and sexes (figure 1A). In Dhaka, where the diversity of serovars and genotypes was greatest, *S* Paratyphi A prevalence was higher in older age groups (44 [32%] of 139 in people aged ≥15 years *vs* 12 [11%] of 105 in children aged <5 years; p=1·6 × 10^–4^ using multivariable logistic regression; figure 1B; appendix 1 p 15), as was the prevalence of non-H58 *S* Typhi and *S* Paratyphi A genotypes (99 [71%] of 139 *vs* 49 [47%] of 105; p=0·010; appendix 1 p 7). Across all sites, 93 cases were considered severe (with symptom duration >10 days or requiring hospitalisation), all of which were caused by *S* Typhi. Severe cases were less common in Kathmandu (13 [10%] of 124) than in Dhaka (62 [17%] of 357) and Blantyre (18 [13%] of 141); figure 1C; appendix 1 p 16). Severe disease was not significantly associated with patient age group or sex, *S* Typhi genotype (H58 *vs* other), or MDR in logistic regression models (figure 1C; appendix 1 p 16).

At each STRATAA site, all local pathogen variants cocirculated throughout the surveillance period (appendix 1 p 6). The dominant genotypes detected in each setting matched those identified in earlier studies,^2^^,^^3^^,^^9^ and contextualisation with global genomes supports that most cases were derived from locally established pathogen variants that are now endemic in their respective settings (appendix 1 p 8). The exception was a cluster of *S* Typhi genotype 3.3.2 in Kathmandu, which appears to have been imported from elsewhere in south Asia, with ancestral sequences isolated from Bangladesh (appendix 1 pp 9–10). This 3.3.2 cluster was first isolated in the Kathmandu STRATAA catchment in February, 2017, and increased, peaking in November, 2017 (nine [90%] of ten monthly *S* Typhi cases) and December, 2017 (six [86%] of seven cases), before declining (appendix 1 pp 9–10).

Genetic mechanisms of resistance are summarised in table 2. Acquired resistance genes for first-line drugs, associated with MDR, were detected in *S* Typhi 4.3.1.1 from Blantyre and Dhaka. The plasmid-borne quinolone-resistance gene *qnrS* was detected in *S* Typhi 4.3.1.3.Bdq in Dhaka, and mutations in the quinolone resistance-determining region (QRDR; associated with non-susceptibility to fluoroquinolones) were common across all *S* Typhi and *S* Paratyphi A genotypes in Dhaka and Kathmandu (not associated with age group or sex; appendix 1 p 7), but were present in just three isolates of *S* Typhi genotype 4.3.1.1.EA1 in Blantyre. The azithromycin resistance-associated^24^ substitution AcrB Arg717Gln was detected in two *S* Typhi and three *S* Paratyphi A isolates from Dhaka.

**Table 2:** Distribution of AMR determinants in *Salmonella enterica* serovars Typhi and Paratyphi A

	Frequency	Acquired genes for first-line drug resistance∗	QRDR or *qnrS*† (fluoroquinolone resistance)	AcrB Arg717Gln (azithromycin resistance)	No AMR
**Blantyre**					
*S* Typhi	141	138/141 (98%)	1 QRDR: 3/141 (2%)	0/141 (0%)	3/141 (2%)
4.3.1.1.EA1	138/141 (98%)	138/138 (100%)	1 QRDR: 3/138 (2%)	0/138 (0%)	0/138 (0%)
Rare genotypes	3/141 (2%)	0/3 (0%)	0/3 (0%)	0/3 (0%)	3/3 (100%)
**Dhaka**					
*S* Typhi	357	182/357 (51%)	1 QRDR: 344/357 (96%); 1 QRDR plus *qnrS*: 12/357 (3%)	2/357 (1%)	1/357 (<1%)
2.0.1	31/357 (9%)	0/31 (0%)	1 QRDR: 31/31 (100%)	0/31 (0%)	0/31 (0%)
2.1.7	27/357 (8%)	0/27 (0%)	1 QRDR: 27/27 (100%)	0/27 (0%)	0/27 (0%)
2.3.3	64/357 (18%)	0/64 (0%)	1 QRDR: 64/64 (100%)	0/64 (0%)	0/64 (0%)
3.2.2	25/357 (7%)	0/25 (0%)	1 QRDR: 25/25 (100%)	1/25 (4%)	0/25 (0%)
4.3.1.1	177/357 (50%)	170/177 (96%)	1 QRDR: 177/177 (100%)	1/177 (1%)	0/177 (0%)
4.3.1.2	2/357 (1%)	0/2 (0%)	1 QRDR: 1/2 (50%); 3 QRDR: 1/2 (50%)	0/2 (0%)	0/2 (0%)
4.3.1.3.Bdq	12/357 (3%)	12/12 (100%)	1 QRDR plus *qnrS*†: 12/12 (100%)	0/12 (0%)	0/12 (0%)
Rare genotypes	19/357 (5%)	0/19 (0%)	1 QRDR: 18/19 (95%)	0/19 (0%)	1/19 (5%)
*S* Paratyphi A	95	0/95 (0%)	1 QRDR: 95/95 (100%)	3/95 (3%)	0/95 (0%)
2.3.2	11/95 (12%)	0/11 (0%)	1 QRDR: 11/11 (100%)	0/11 (0%)	0/11 (0%)
2.3.3	23/95 (24%)	0/23 (0%)	1 QRDR: 23/23 (100%)	0/23 (0%)	0/23 (0%)
2.4.4	56/95 (59%)	0/56 (0%)	1 QRDR: 56/56 (100%)	3/56 (5%)	0/56 (0%)
Rare lineages	5/95 (5%)	0/5 (0%)	1 QRDR: 5/5 (100%)	0/5 (0%)	0/5 (0%)
**Kathmandu**					
*S* Typhi	124	2/124 (2%)	1 QRDR: 106/124 (85%) 3 QRDR: 3/124 (2%)	0/124 (0%)	14/124 (11%)
3.3.2	41/124 (33%)	0/41 (0%)	1 QRDR: 31/41 (76%)	0/41 (0%)	10/41 (24%)
4.3.1	10/124 (8%)	0/10 (0%)	1 QRDR: 10/10 (100%)	0/10 (0%)	0/10 (0%)
4.3.1.1	3/124 (2%)	2/3 (67%)	1 QRDR: 2/3 (67%)	0/3 (0%)	0/3 (0%)
4.3.1.2	67/124 (54%)	0/67 (0%)	1 QRDR: 63/67 (94%); 3 QRDR: 3/67 (4%)	0/67 (0%)	1/67 (2%)
Rare genotypes	3/124 (2%)	0/3 (0%)	0/3 (0%)	0/3 (0%)	3/3 (100%)
*S* Paratyphi A	14	0/14 (0%)	1 QRDR: 14/14 (100%)	0/14 (0%)	0/14 (0%)
Rare lineages	14/14 (100%)	0/14 (0%)	1 QRDR: 14/14 (100%)	0/14 (0%)	0/14 (0%)

Data are n/N (%); percentages represent proportion of serovar (for “Frequency” column) or proportion of the corresponding genotype or lineage (remaining columns). Data represent the main STRATAA dataset (n=622 *S* Typhi and n=109 *S* Paratyphi A; table 1). Genotypes with ≥10 samples or representing a major 4.3.1 (H58) sublineage are shown; the remaining genotypes per site are grouped as “rare genotypes”. AMR=antimicrobial resistance. QRDR=quinolone resistance-determining region. 1 QRDR=one QRDR mutation (associated with decreased ciprofloxacin susceptibility). 3 QRDR=three QRDR mutations (associated with ciprofloxacin resistance).

∗ All acquired genes for resistance to first-line drugs (ie, chloramphenicol, co-trimoxazole, and ampicillin) were associated with chromosomally integrated transposons.

† 4.3.1.1.Bdq genomes carried *blaTEM-1*, *sul2*, and *tet(A)* together with *qnrS* in an IncFIB_K_ plasmid.

Resistance genes were almost exclusively integrated into the *S* Typhi chromosome, most frequently mediated by the translocation of a Tn*2670*-like composite transposon (carrying genes *catA1*, *dfrA7*, *bla*_TEM-1_, *strAB*, *sul1*, and *sul2*; 279 [38%] of 731 genomes in the main surveillance periods), and occasionally by transposon Tn*2670* (*catA1*, *dfrA7*, and *sul1*; 27 [4%] genomes) or Tn*6029* (*bla*_TEM-1_, *strAB*, *sul2*; one [<1%] genome). The exception to this was *qnrS*, which was carried by an IncFIB_K_ plasmid (also carrying genes *bla*_TEM-1_, *sul2*, *tet(A)*; 12 [2%] genomes). In Dhaka, MDR *S* Typhi was significantly less common in adults (OR 0·48 [95% CI 0·26-0·87] for age ≥15 years compared with age <5 years, p=0·020; appendix 1 p 7).

Antimicrobial susceptibility phenotypes were determined by disc-diffusion^20^ for a subset of isolates, and non-susceptibility was well predicted by known determinants (in *S* Typhi, 99% positive predictive value for first-line drugs and >96% for ciprofloxacin; in *S* Paratyphi A, 99% positive predictive value for ciprofloxacin; appendix 1 pp 17–18). Phenotypic assessment of azithromycin susceptibility is challenging^25^ and susceptibility thresholds are poorly defined. Three of five *acrB* mutants tested showed resistance to azithromycin. Several other isolates showed azithromycin-resistant phenotypes, but had wild-type *acrB* genes and 23S alleles, no acquired macrolide-resistance genes, and genome-wide screening did not identify any novel variants (appendix 1 pp 4–5).

We used ancestral state reconstruction of AMR determinants on global phylogenies to differentiate the emergence of AMR from transmission of resistant strains (table 3). Overall, most enteric fever cases in all three study sites were due to circulation of locally established pathogen variants, and AMR infections were overwhelmingly caused by transmission of pre-existing AMR strains (702 [98%] of 713 cases). In Blantyre, all MDR cases were attributed to local transmission of MDR *S* Typhi 4.3.1.1.EA1 (appendix 1 p 11).^9^ By contrast, all three QRDR mutations in Blantyre were attributed to local evolution, arising independently in endemic MDR *S* Typhi 4.3.1.1.EA1, with no evidence of transmission to secondary cases (appendix 1 p 11).

**Table 3:** AMR transmission

	Resistant cases	Clusters	Source of resistance		
			Transmitted local variant	Transmitted imported variant	De novo evolved variant
**Blantyre**					
*S* Typhi					
First-line drug-resistance gene	138/141 (98%)	1	138/138 (100%)	0/138 (0%)	0/138 (0%)
1 QRDR	3/141 (2%)	3	0/3 (0%)	0/3 (0%)	3/3 (100%)
**Dhaka**					
*S* Typhi					
First-line drug-resistance gene	170/357 (48%)	2	170/170 (100%)	0/170 (0%)	0/170 (0%)
1 QRDR∗	343/357 (96%)	13	343/343 (100%)	0/343 (0%)	0/343 (0%)
3 QRDR	1/357 (<1%)	1	0/1 (0%)	1/1 (100%)	0/1 (0%)
QRDR and *qnrS*	12/357 (3%)	1	12/12 (100%)	0/12 (0%)	0/12 (0%)
AcrB Arg717Gln†	2/357 (1%)	2	1/2 (50%)	0/2 (0%)	1/2 (50%)
*S* Paratyphi A					
1 QRDR	95/95 (100%)	4	94/95 (99%)	0/95 (0%)	1/95 (1%)
AcrB Arg717Gln†	3/95 (3%)	2	2/3 (67%)	0/3 (0%)	1/3 (33%)
**Kathmandu**					
*S* Typhi					
First-line drug-resistance gene	2/124 (2%)	2	2/2 (100%)	0/2 (0%)	0/2 (0%)
1 QRDR	106/124 (85%)	8	103/106 (97%)	1/106 (1%)	2/106 (2%)
3 QRDR	3/124 (2%)	1	3/3 (100%)	0/3 (0%)	0/3 (0%)
*S* Paratyphi A					
1 QRDR	14/14 (100%)	3	13/14 (93%)	1/14 (7%)	0/14 (0%)

Data are n/N (%). Each row represents a specific AMR pattern identified in a given *Salmonella enterica* serovar (*S* Typhi or *S* Paratyphi A) at a given site, and summarises the number of cases sequenced, the number of independent lineages those cases cluster into in the phylogeny, and the number of resistant cases attributed to transmission of local or imported resistant variants or to de novo evolution of resistance. The three source categories were assigned based on ancestral state reconstruction of AMR determinants and country on the global-context phylogenies (appendix 1 p 5). AMR=antimicrobial resistance. QRDR=quinolone resistance-determining region. 1 QRDR=one QRDR mutation (associated with decreased ciprofloxacin susceptibility). 3 QRDR=three QRDR mutations (associated with ciprofloxacin resistance).

∗ Excluding cases with *qnrS* mutations.

† *acrB* mutation associated with azithromycin resistance.

In Dhaka, resistance to first-line drugs was attributed to local transmission of *S* Typhi 4.3.1.1 (MDR) and 4.3.1.3.Bdq (ampicillin-resistant) that had become endemic before the surveillance period (appendix 1 p 12).^2^^,^^7^ Nearly all sequences carrying QRDR mutations in Dhaka (n=356 *S* Typhi, n=95 *S* Paratyphi A) were attributed to local transmission of endemic strains (table 3; appendix 1 p 12). The exceptions were one case of *S* Paratyphi A 2.4.4 (appendix 1 pp 9–10; de novo evolution), and one of *S* Typhi 4.3.1.2, probably imported from India^3^^,^^7^^,^^10^ (appendix 1 pp 9–10). The other ciprofloxacin-resistant *S* Typhi cases in Dhaka were attributed to local transmission of endemic strain 4.3.1.3.Bdq (appendix 1 p 12). AcrB Arg717Gln mutations were either inherited from local populations (one case of *S* Typhi 4.3.1.1; two cases of *S* Paratyphi A 2.4.4; appendix 1 pp 9–10), or arose independently in local populations (one case of *S* Typhi 3.3.2; one case of *S* Paratyphi A 2.4.4; appendix 1 pp 9–10).

In Kathmandu, resistance to former first-line drugs chloramphenicol, co-trimoxazole, and ampicillin was rare (two [1%] of 138 cases), occurred only in *S* Typhi 4.3.1.1 (appendix 1 p 13), and resulted from local transmission of endemic strains pre-dating STRATAA surveillance (table 3). Most cases (106 [85%] of 124 *S* Typhi and all *S* Paratyphi A) carried a single QRDR mutation. These cases resulted from 11 different clusters, each with a *gyrA* mutation (appendix 1 p 13), most of which (103 [97%] of 106 of *S* Typhi, 13 [93%] of 14 *S* Paratyphi A) were already present in the local population and carrying QRDR mutations before STRATAA surveillance (table 3). The exceptions were one case of *S* Paratyphi A 2.3 (appendix 1 pp 9–10) and one of *S* Typhi 4.3.1.1 (appendix 1 pp 9–10), closely related to sequences from India (8–9 SNVs); and two *S* Typhi 3.2.2 with no close relatives (appendix 1 pp 9–10).

**Figure 2:**
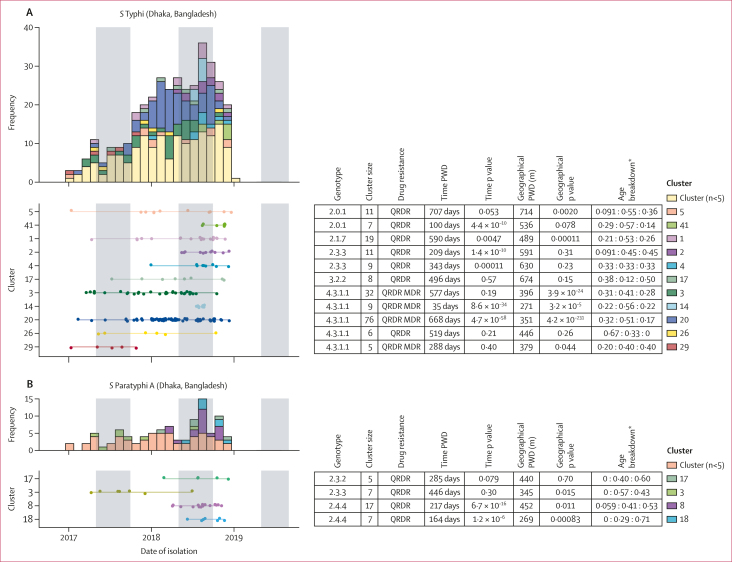

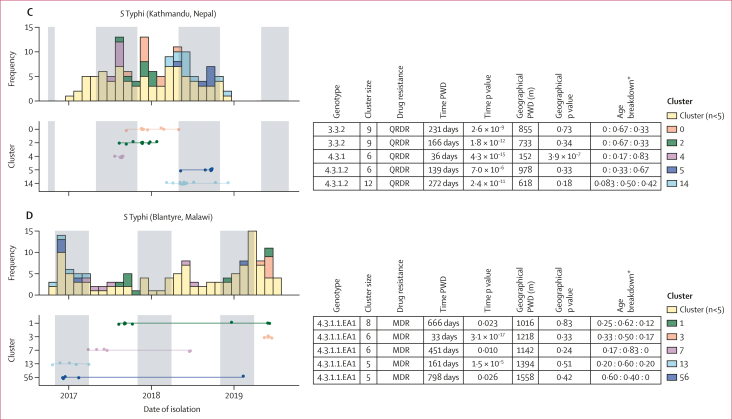
Features of zero-SNV clusters of *Salmonella enterica* serovars Typhi and Paratyphi A Each panel summarises clusters for *S* Typhi in Dhaka (A), *S* Paratyphi A in Dhaka (B), *S* Typhi in Kathmandu (C), and *S* Typhi in Blantyre (D). Data are shown for common zero-SNV clusters (comprising at least five cases). Left panels show monthly counts for each cluster (upper panel) above a timeline, with one row per cluster, as labelled (lower panel). Darker shaded vertical areas in the plots represent the rainy season in each location. Tables summarise key information per cluster (rows as per timeline figure); colour key indicates colour code per cluster, which applies across the whole panel. Time p value and geographical p values are from Kolmogorov–Smirnov tests comparing the pairwise distribution within a given cluster to the distribution between all unclustered isolates from that site. PWD=pairwise distance. ∗Age breakdown shows ratios of participants aged <5 years to those aged 5–15 years to those aged ≥15 years.

Substitution mutations accumulate too slowly in *S* Typhi and *S* Paratyphi A to infer specific transmission events from sequence data.^26^ Therefore, instead of using complex phylodynamic transmission-chain reconstruction methods to explore transmission patterns, we examined groups of cases that formed zero-SNV clusters (ie, no SNVs detected in the core genome), which we interpret as being linked either by a common source or by chains of transmission during which no substitutions have arisen. 479 (66%) of 731 cases fell into zero-SNV clusters (85 [60%] of 141 in Blantyre, 313 [69%] of 452 in Dhaka, and 81 [59%] of 138 in Kathmandu). Most clusters had two cases (40 clusters) or three cases (22 clusters), but there were also 25 large clusters of five or more cases (30 [21%] of 141 cases in Blantyre, 229 [51%] of 452 in Dhaka, and 42 [30%] of 138 in Kathmandu). The median time between consecutive cases in the same cluster was 16 days (IQR 3–49 days). Median time between first and last cases per cluster was 136 days, or 4·5 months (37–273 days, or 1·2–9·1 months) and the maximum was 854 days (28 months, or 2·3 years). Zero-SNV clusters spanning more than 1 year were detected in all sites. This finding confirms the slow substitution rate and highlights the difficulty in resolving individual transmission events for typhoidal pathogens. Cases from the same zero-SNV clusters showed significant temporal clustering (reduced pairwise temporal distances compared with unclustered isolates; figure 2) in all settings and for both serovars. From 648 (89%) of 731 cases with available GPS coordinates (427 [94%] of 452 in Dhaka, 107 [78%] of 138 in Kathmandu, and 114 [81%] of 141 in Blantyre), we identified evidence of geographical clustering of zero-SNV cases compared with unclustered cases in south Asian settings but not in Blantyre (median distance 373 m *vs* 540 m for *S* Typhi in Dhaka; 421 m *vs* 524 m for *S* Paratyphi A in Dhaka; and 646 m *vs* 796 m for *S* Typhi in Kathmandu; too few *S* Paratyphi A samples were available for analysis in Kathmandu; appendix 1 p 14). Figure 2 shows properties of the 25 larger clusters (five or more cases). Ten of these clusters showed evidence of spatial clustering, as expected for transmission following a point-source outbreak: nine in Dhaka (*S* Typhi 2.0.1, 2.1.7, and 4.3.1.1, spanning up to ∼2 years each; *S* Paratyphi A 2.3.3 and 2.4.4, ∼14 months) and one in Kathmandu (*S* Typhi 4.3.1, 36 days; figure 2).

AMR profiles were homogeneous within clusters (figure 2), consistent with transmission of AMR variants. In Kathmandu, cases caused by the largely imported strain *S* Typhi 3.3.2 were more likely to be in zero-SNV clusters (29 [71%] of 41, in six clusters) compared with endemic *S* Typhi 4.3.1.2 (41 [61%] of 67, 11 clusters), other *S* Typhi (six [38%] of 16, one cluster), or *S* Paratyphi A (five [36%] of 14, two clusters; p=0·030, χ^2^ test). In Dhaka, some genotypes had significantly higher clustering rates (146 [82%] of 177 for genotype 4.3.1.1; nine [75%] of 12 for 4.3.1.3; and 21 [68%] of 31 for 2.0.1) than others (38 [59%] of 64 for 2.3.3; 59 [62%] of 95 for *S* Paratyphi A; p<1x10^–6^, χ^2^ test), which might reflect greater intensity of transmission.^20^ In all settings, all age groups were equally likely to be in zero-SNV clusters (160 [64%] of 249 in age <5 years, 226 [66%] of 340 in age 5–15 years, 93 [65%] of 142 in age ≥15 years; p>0·4 in all settings, χ^2^ test), and most clusters (73 [77%] of 95) were detected across age groups (figure 2).

## Discussion

This study provides a comprehensive view of pathogen populations underlying enteric fever in three urban sites in areas of high burden. Use of the same surveillance protocols across all sites, in defined catchments and including all ages, allows the results to be compared between sites. Genotype prevalences were in line with other studies reported from the same cities,^2^^,^^3^^,^^7^, ^8^, ^9^, ^10^^,^^21^^,^^27^^,^^28^ but we also specifically addressed pathogen diversity across age groups. Although disease burden is highest in children,^20^ we found that the same pathogen variants (serovars, genotypes, and AMR) circulate across age groups (pre-school, school-age, and adult), with near-identical sequences (zero-SNV clusters) identified across age groups, supporting the assumption of homogeneous mixing between age groups in models of TCV impact. These findings also support the idea that vaccinating children might have a secondary impact by reducing transmission to adults, although the directionality of transmission is not resolvable from WGS, and no statistically significant effect has been observed in TCV trials.^12^, ^13^, ^14^, ^15^, ^16^, ^17^, ^18^

Importantly, this study explicitly addresses the transmission burden of drug resistance in enteric fever. Several previous studies have documented transmission of AMR variants between countries, including the introduction of MDR *S* Typhi 4.3.1 variants from south Asia into Kenya, Malawi, and neighbouring countries,^8^^,^^9^^,^^29^ and the spread of ciprofloxacin-resistant *S* Typhi 4.3.1.2 from India into Nepal.^3^^,^^10^ A previous study estimated that QRDR mutations have arisen at least 94 times in *S* Typhi and transferred between countries at least 119 times.^7^ It is also frequently reported that AMR infections result from clonal spread. However, although several studies have used WGS and ancestral state reconstruction to estimate the proportion of AMR *Mycobacterium tuberculosis* infections that are due to the transmission of AMR variants versus de novo emergence,^30^ such a proportion has not been quantified for enteric fever pathogens. Our results show that most enteric fever cases in all three study sites were due to circulation of locally established pathogen variants, and that AMR infections were overwhelmingly caused by transmission of pre-existing AMR strains (702 [98%] of 713 cases). This finding supports the modelling assumption that each AMR infection poses a risk of secondary infections in others through shedding and onward transmission. Coupled with our data showing that the same variants cocirculate across age groups, this finding lends weight to model predictions that reducing the incidence of AMR *S* Typhi infection and shedding in children through TCVs might have a secondary effect of reducing exposure to AMR infections across the population.

Our data showing persistence of zero-SNV clusters over months and years highlight the challenges of inferring transmission events from *S* Typhi WGS data. Previous studies have estimated substitution rates of approximately one SNV every 2 years,^3^^,^^8^ a log-scale slower than for host-generalist *S enterica* serovars such as Agona and Kentucky, and similar to slow-growing *M tuberculosis*.^31^ Our data concur with previous assertions that the evolutionary rate in *S* Typhi is too slow to support the inference of explicit transmission events and networks from WGS data.^26^ Although this limitation prevents reconstruction of transmission chains, it is still possible to obtain useful data on transmission dynamics from WGS data (eg, R_0_ and trends in effective population size).^7^ Spatial analysis using GPS coordinates without topological mapping identified significant spatial clustering in Dhaka and Kathmandu, but not in Blantyre. The Blantyre area has a complex river system, comprising ten catchments in close proximity, and recent spatial modelling of *S* Typhi in this setting showed that dividing space into river catchments explained the spatiogenetic patterns better than grid coordinates.^28^ Detailed spatial modelling is beyond the scope of this study, but is planned for all sites.

The main limitations of this study were the low blood culture sensitivity (∼50%), and sequencing of isolates from a single urban catchment per country; therefore, our findings might not be representative of country-wide populations. Moreover, some cases were unable to be sequenced due to logistical challenges. Consequently, future surveillance studies including additional geographical locations, with real-time in-country pathogen sequencing to ensure inclusion of all blood culture isolates, would be valuable to gain deeper insights into country-specific trends in pathogen transmission dynamics.

In summary, our findings strengthen efforts to model TCV impact and provide baseline data for assessing the impact of future immunisation programmes and other interventions. The data also highlight the ongoing spread of AMR typhoid throughout all three settings, and underscore the need for improvements to water sanitation and hygiene infrastructure and the introduction of TCVs to improve typhoid control.

## Data sharing

All raw whole-genome sequencing data have been deposited in the European Nucleotide Archive (project accession number PRJEB14050). Individual accession numbers and all other data underlying this study have been provided in appendix 2. Data are available freely and indefinitely.

## Declaration of interests

AJP is Chair of the UK Government Department of Health and Social Care’s Joint Committee on Vaccination and Immunisation (unpaid) and was a member of WHO's SAGE until 2022 (unpaid). VEP has received travel reimbursement from Merck and Pfizer for attending scientific input engagements unrelated to the topic of the manuscript, and is a member of the WHO Immunization and Vaccine-related Implementation Research Advisory Committee. All other authors declare no competing interests.
