# Supplementary material for: Pathogen diversity and antimicrobial resistance transmission of Salmonella enterica serovars Typhi and Paratyphi A in Bangladesh, Nepal, and Malawi: a genomic epidemiological study
Source: Lancet Microbe. 2024 Aug;5(8):None. doi: 10.1016/S2666-5247(24)00047-8 (PMC11300424; doi:10.1016/S2666-5247(24)00047-8)
Supplement: Supplementary appendix 1 [file mmc1.pdf]

THE LANCET
Microbe

Supplementary appendix 1

This appendix formed part of the original submission and has been peer reviewed.
We post it as supplied by the authors.

Supplement to: Dyson ZA, Ashton PM, Khanam F, et al. Pathogen diversity and antimicrobial resistance transmission of *Salmonella enterica* serovars Typhi and Paratyphi A in Bangladesh, Nepal, and Malawi: a genomic epidemiological study. *Lancet Microbe* 2024. https://doi.org/10.1016/S2666-5247(24)00047-8

Supplementary Material

Supplementary Methods

Supplementary Figures

Figure S1: Genetic diversity of enteric fever pathogen populations.

Figure S2: Breakdown of patient sex and Typhi features, within each age-group, at each site, for the main STRATAA dataset.

Figure S3: Phylogenetic trees of Typhi and Paratyphi A showing STRATAA isolate sequences in context with publicly available global genome data.

Figure S4: Detail of subpopulations discussed in text.

Figure S5: Phylogenetic tree of Typhi sequenced from STRATAA surveillance in Blantyre.

Figure S6: Phylogenetic tree of Typhi sequenced from STRATAA surveillance in Dhaka.

Figure S7: Phylogenetic tree of Typhi sequenced from STRATAA surveillance in Kathmandu.

Figure S8: Spatial distribution of zero-SNV clusters from STRATAA surveillance

Supplementary Tables

Table S1: Typhi and Paratyphi A genomes sequenced in this study
[CSV file]

Table S2: Logistic regression models testing for association of age and sex with pathogen features.

Table S3: Logistic regression models of severe Typhi infection.

Table S4: Comparison of phenotypic and genotypic assessment of non-susceptibility to antimicrobials for Typhi.

Table S5: Comparison of phenotypic and genotypic assessment of non-susceptibility to antimicrobials for Paratyphi A.

Table S6: Publicly available Typhi and Paratyphi A genome sequences used to provide global context for genotypes detected in STRATAA sequences
[CSV file]

Table S7: Representative Typhi sequences (one per defined genotype) used to outgroup-root Typhi phylogenies
[CSV file]

Supplementary Methods

Passive surveillance for enteric fever

The manuscript reports genome sequence data for all *Salmonella* isolates collected during passive surveillance for enteric fever during the STRATAA study. The full STRATAA protocol is reported in Darton *et al.* 2017[1] and the primary disease burden results are reported in Meiring *et al* . 2022[2]; key details are summarised here.

The three study sites in Blantyre (Malawi), Dhaka (Bangladesh), and Kathmandu (Nepal) were selected from locations across Africa and Asia based on known high rates of enteric fever and capacity to deliver a large-scale and logistically complexity study. Each study site demonstrated different epidemiological profiles and historical patterns of enteric fever incidence[1]. Sample size calculations are described in the STRATAA protocol, and the target catchment population size (n=100,000) was powered to estimate typhoid incidence rate through passive surveillance with precision (half-width) of 50%[1]. Baseline and final demographic censuses enumerated a target population around 100,000 at each site including 22,000-26,000 households[2]. Participants residing in these catchment areas were encouraged to attend study health-care facilities for blood culture and treatment if they developed fever. Those presenting with a history of fever for ≥48 h or current temperature of ≥38·0°C were approached for recruitment. For analyses presented here, the inclusion criteria was blood culture confirmed cases derived from separate individuals resident within the census area, collected within the surveillance period. For Dhaka and Kathmandu the surveillance period was Jan 2017–Dec 2018, and Oct 2016–Sep 2018 for Blantyre. As detailed in Table 1, an additional ten months of surveillance data was available for Blantyre, which was leveraged to better understand these populations, but these data were not included in diversity analyses to prevent bias from a longer sampling frame.

Bacterial isolates and whole genome sequencing

Isolates cultured from blood of febrile individuals recruited into the STRATAA passive surveillance studies at each of the three sites were stored locally until the end of the recruitment period. Antimicrobial susceptibility testing was performed by disk diffusion method for Ampicillin, Azithromycin, Ceftriaxone, Cefixime, Ciprofloxacin, Chloramphenicol, Co-trimoxazole, Nalidixic Acid and Meropenem following either Clinical and Laboratory Standards Institute (CLSI)[3] or British Society of Antimicrobial Chemotherapy (BSAC) breakpoint guidelines (available at: http://www.bsac.org.uk/) as described in reference 2. Subsequently, isolates were cultured overnight and genomic DNA extracted using the Wizard Genomic DNA Extraction Kit following the manufacturers recommendations (Promega, WI, USA). DNA was shipped to the Wellcome Sanger Institute and subjected to indexed whole genome sequencing on an Illumina HiSeq 2500 platform to generate paired-end reads of 100 bp in length, as described previously[4].

Isolates from n=452/454 cases in Dhaka (99·6% of all culture-positive) from the period January 2017-December 2018 were successfully sequenced and confirmed as Typhi or Paratyphi A (summary in Table 1, genome list in **Table S1**). From Kathmandu, n=138/164 cases (84·1% of all culture-positive) from the same time period were successfully sequenced and serovars confirmed. From Blantyre, n=83/115 cases (72·2% of all culture-positive) from October 2016-October 2018 were successfully sequenced and typhoidal serovars confirmed. Recruitment

continued for an additional 10 months in Blantyre, resulting in total n=141/158 sequenced typhoidal isolates (89·2% of positive cultures). The full set of n=141 sequences from Blantyre are included in the main dataset 'STRATAA', which comprises genome sequence data for n=731 unique typhoidal *Salmonella* blood-culture isolates (622 Typhi and 109 Paratyphi A, see **Table 1**). A small number of additional blood-culture isolates were captured at the study sites before or after these formal surveillance periods (n=30 from Dhaka, n=27 from Kathmandu) or outside the boundaries of the surveillance catchment area (n=35 from Blantyre); these were also sequenced and included in phylogenetic trees to provide context (total n=707 Typhi, n=116 Paratyphi A; see **Table S1**), but were excluded from statistical analyses.

Single nucleotide variant (SNV) analysis and *in silico* genotyping

Raw Typhi Illumina reads were mapped to the Typhi CT18 reference sequence (accession no. AL513382)[5], and those for Paratyphi A to the ParatyphiA AKU_12601 reference genome (accession no. FM200053)[6]. Mapping was carried out using the RedDog mapping pipeline (V1beta.11; available at https://github.com/katholt/reddog), which uses Bowtie (v2.2.9)[7] to map reads to the reference sequence, and SAMtools (v1.3.1)[8] to identify single nucleotide variant (SNV) calls as previously described[9]. All raw read data analysed had a minimum read depth of >40-fold, and >97% reference coverage. For Typhi sequences, read alignments (BAM files) were then used as input for GenoTyphi (v1.9.1; available at: https://github.com/katholt/genotyphi)[10] to assign Typhi isolates to known genotypes according to an extended Typhi genotyping framework[10,11]. Similarly, Paratype (v1.0; available at https://github.com/CHRF-Genomics/Paratype) was used to assign Paratyphi A sequences to genotypes[12].

Chromosomal SNVs with confident homozygous base calls (phred score >20), for all SNV sites that had such calls in >95% of Typhi genomes (representing the 95% 'soft' core genome) were concatenated to form an alignment of alleles at 15,387 variant sites for all Typhi, including 707 from this study (Table S1) and 3,128 global context sequences (Table S6) from previous studies[9,13–22]. Previously defined[11,23,24] repetitive and recombinant regions were excluded (354 kb; ~7·4% of bases in the CT18 reference chromosome), and any remaining recombination filtered out using Gubbins (v2.4.1)[25] resulting in a final alignment length of 14,780 chromosomal SNVs. Typhi phylogenies were outgroup rooted using Paratyphi A AKU_12601 alleles, and a representative selection of Typhi outgroup taxa from each defined genotype (Table S7). An interactive version of the Typhi phylogeny with STRATAA plus global isolates (shown in Fig.S3) is available at https://microreact.org/project/wim5TssQ3AqSfgWXTSP2Bd. Genotype-specific phylogenies were constructed in the same manner (used for ancestral state reconstruction, described below); as were local site-specific phylogenies of STRAATA data (Figs.S5-S7), and Paratyphi A phylogenies (outgroup-rooted using Typhi CT18 alleles, https://microreact.org/project/SgHbIR9cP).

Phylogenomic analysis

Maximum-likelihood (ML) phylogenetic trees were inferred from the aforementioned chromosomal SNV alignments using RAxML (v8.2.8)[26]. A generalised time-reversible model and a Gamma distribution was used to model site-specific rate variation (GTR+Γ substitution model; GTRGAMMA in RAxML) with 100 bootstrap

pseudoreplicates[27] used to assess branch support for the ML phylogeny. The resulting phylogenies were visualised and annotated using Microreact[28] and the R package *ggtree* (v2.2.4)[29] .

Identification of antimicrobial resistance (AMR) determinants and associated mobile genetic elements

For the detection of chromosomal point mutations associated with AMR, GenoTyphi (v1.9.1; available at: https://github.com/katholt/genotyphi)[10,11] and GenoParatyphi (v0.1-alpha; available at: https://github.com/zadyson/genoparatyphi) were used. These tools screen for mutations in the quinolone resistance determining region (QRDR) of *gyrA* (codons 83 and 87), *parC* (codons 80 and 84) and *gyrB* (codon 464), and mutations at *acrB* codon 717 (azithromycin resistance)[30,31]. Acquired genes and plasmid replicons were detected using the mapping-based allele typer SRST2 (v0.2.0)[32] to screen against the ARG-ANNOT[33] and PlasmidFinder[34] databases, respectively.

Comparison of AMR genotypes and phenotypes

To assess how well genetic determinants predict AMR phenotypes, we interpreted the following markers as predictive of non-susceptibility to specific antimicrobials, based on previous publications[31,35]: $bla_{\text{TEM-1}}$, ampicillin; carbapenemase, meropenem; *catA1*, chloramphenicol; *dfr* plus *sul*, co-trimoxazole; extended-spectrum beta-lactamase, ceftriaxone and cefixime; *acrB*-717 mutation or *mphA*, azithromycin; QRDR mutation or *qnrS*, ciprofloxacin and nalidixic acid. Positive predicted values, negative predicted values, and 95% confidence intervals were determined using the *epi.tests()* function in the R package epiR (v2.0.62)[36]. Results are reported for drugs that were tested in ≥20% of isolates.

As most azithromycin resistance was not explained by known mechanisms (*acrB*-717 mutation or acquired genes), we screened Ribosomal RNA (rRNA) operons for potential explanatory mutations. A single copy of the rRNA operon (coordinates 4257263-4262892 in the CT18 reference genome) was extracted, and used as a reference sequence for mapping of Typhi reads using RedDog (as described above). Read alignments (BAM format) were subjected to low-frequency variant calling LoFreq (v2.1.3.1)[37], and SNV read-depth data extracted from the resultant variant calling format (VCF) files with the *read.vcfR*() function from the R package *vcfR* (v1.12.0)[38]. Read depths for SNVs detected in the rRNA operon were normalised using the average chromosomal read depths for each sequence reported by RedDog across the entire CT18 reference sequence to identify single copy mutations (read depth of ~1x the average reported for the CT18 chromosome). These single copy mutations were examined for an association with observed azithromycin resistance phenotypes, but were found to correlate with the genotype backgrounds in which they occurred and not with resistance. The same approach was used to assess Paratyphi A genomes, where reads were mapped to a single rRNA operon (coordinates 3870557-3876131 in the Paratyphi A AKU_12601 reference genome), but again no evidence of resistance-associated mutations in the rRNA operon were identified.

DBGWAS (v0.5.4)[39] was utilised to carry out a bacterial genome-wide association study (GWAS) to screen for genetic loci and/or variants associated with the azithromycin resistance. For this, raw reads were assembled *de novo* with Unicycler (v0.4.7)[40] and used as input to DBGWAS, along with azithromycin resistance status for

each sequence. DBGWAS was run using default parameters and p-value <0·01 interpreted as a significant association; none were identified.

Transmission of molecular determinants of AMR

To determine if resistant enteric fever cases resulted from infection with locally-circulating resistant strains, imported strains, or emerged *de novo*, we carried out ancestral state reconstruction (ASR) analyses. For each genotype where molecular determinants of AMR were detected, we inferred a ML phylogeny including all available STRATAA genomes plus global contextual sequences of the same genotype (phylogenetic inference methods as detailed above). We then conducted ASR, for each detected AMR determinant and country of origin (Nepal, Bangladesh, Malawi, or other), onto these ML tree topologies using a maximum-parsimony method implemented in the *ancestral.pars*() function in the R package *phangorn* (v2.5.5)[41]. The inferred states for each variable (country and AMR determinant) were extracted for each internal node of each tree and used to infer, for each resistant isolate (tree tip), whether the resistance was most likely: (i) inherited from a local resistant strain (parent node and tip share same AMR determinant and location, interpreted as local transmission of a resistant strain); (ii) inherited from an imported resistant strain (parent node and tip share the same AMR determinant but parent node is located in a different country, interpreted as transmission of an imported resistant strain); (iii) recently emerged *de novo* (parent node lacks the AMR determinant, interpreted as recent emergence of resistance rather than transmission of an established resistant strain).

Logistic regression models

Multivariable logistic regression models were fit using the *logistf* package (v1.24.1)[42], which implements Firth's bias-reduced penalized-likelihood logistic regression. Age-group was treated as a categorical variable reflecting groups that have broadly different contact networks relevant to enteric fever transmission/exposure: pre-school age (<5 years and interacting mainly with other household members, reference category in logistic regression), school-age children (5-15 years, interacting with household members and other school-age children), and working age (≥15 years old, interacting with household members and the wider community). Notably, treating age-group categories as ordinal, or treating age (in years) as a continuous variable, gave comparable results in logistic regression models with no significant difference in model fit (assessed with AIC); therefore we report all results using categorical age groups as we consider these more interpretable in terms of enteric fever epidemiology. Other variables (serovar, dominant genotype, MDR, severity) were coded as binary data.

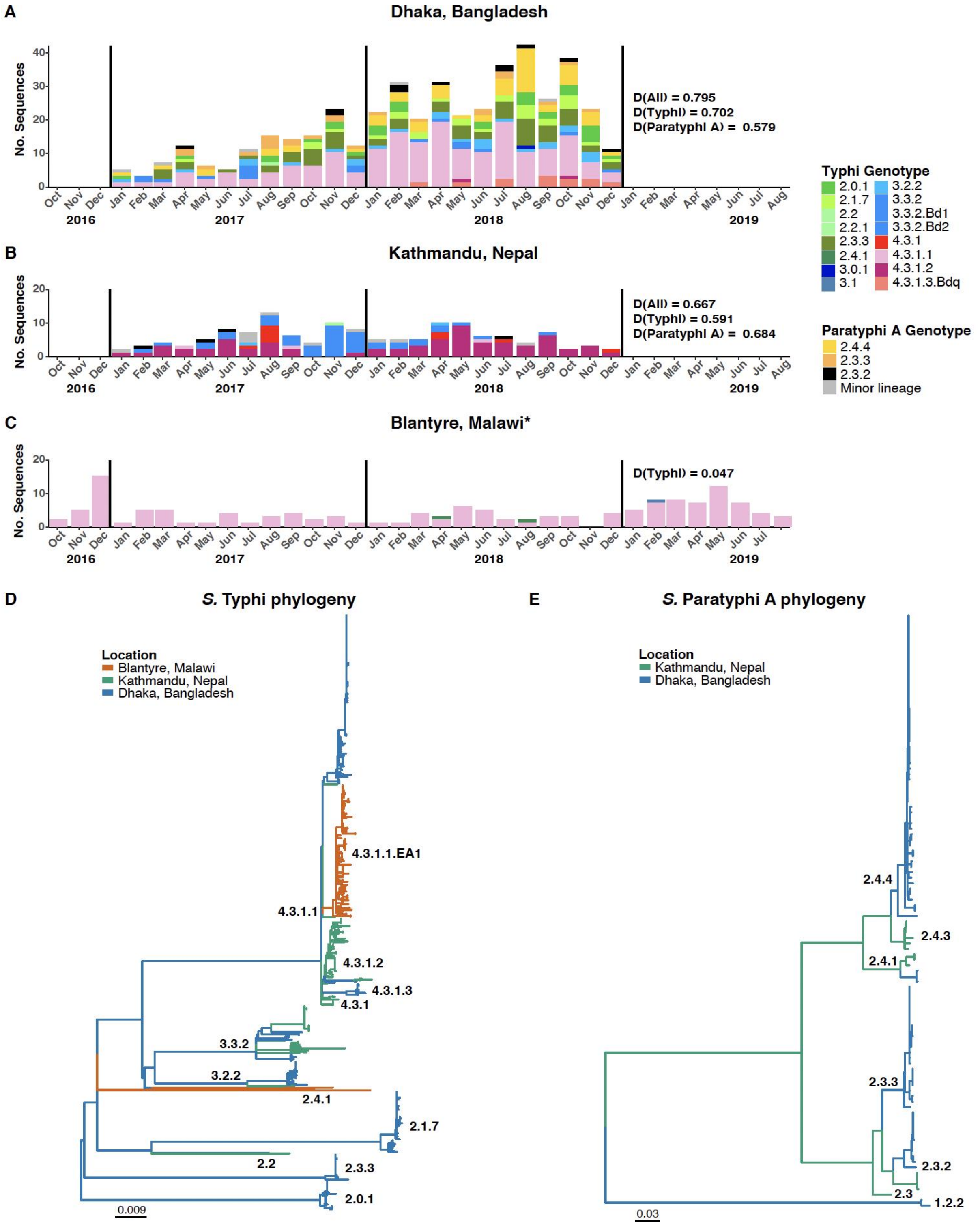

Figure S1. Genetic diversity of enteric fever pathogen populations. (A-C) Epidemic curves for sequenced enteric fever cases in the three STRATAA catchment areas, stratified by pathogen genotype. Bars indicate monthly case counts, coloured to indicate serovar and genotype as per inset legend. *4.3.1.1 in Blantyre is sublineage 4.3.1.1.EA1. Inset labels 'D( )' indicate Simpson's diversity index calculated from pathogen genotype counts for Typhi, Paratyphi A or both ('D(All)'). **(D-E)** Maximum-likelihood phylogenetic trees for STRATAA isolates of serovars Typhi (D) and Paratyphi A (E), inferred from genome-wide single nucleotide variant alignments. Each tree was outgroup-rooted using genomes of the other serovar. Branches are coloured by country as per inset legend, and labelled by genotype. Data in all panels (A-E) represent the main STRATAA dataset (n=622 Typhi and n=109 Paratyphi A; see Table 1).

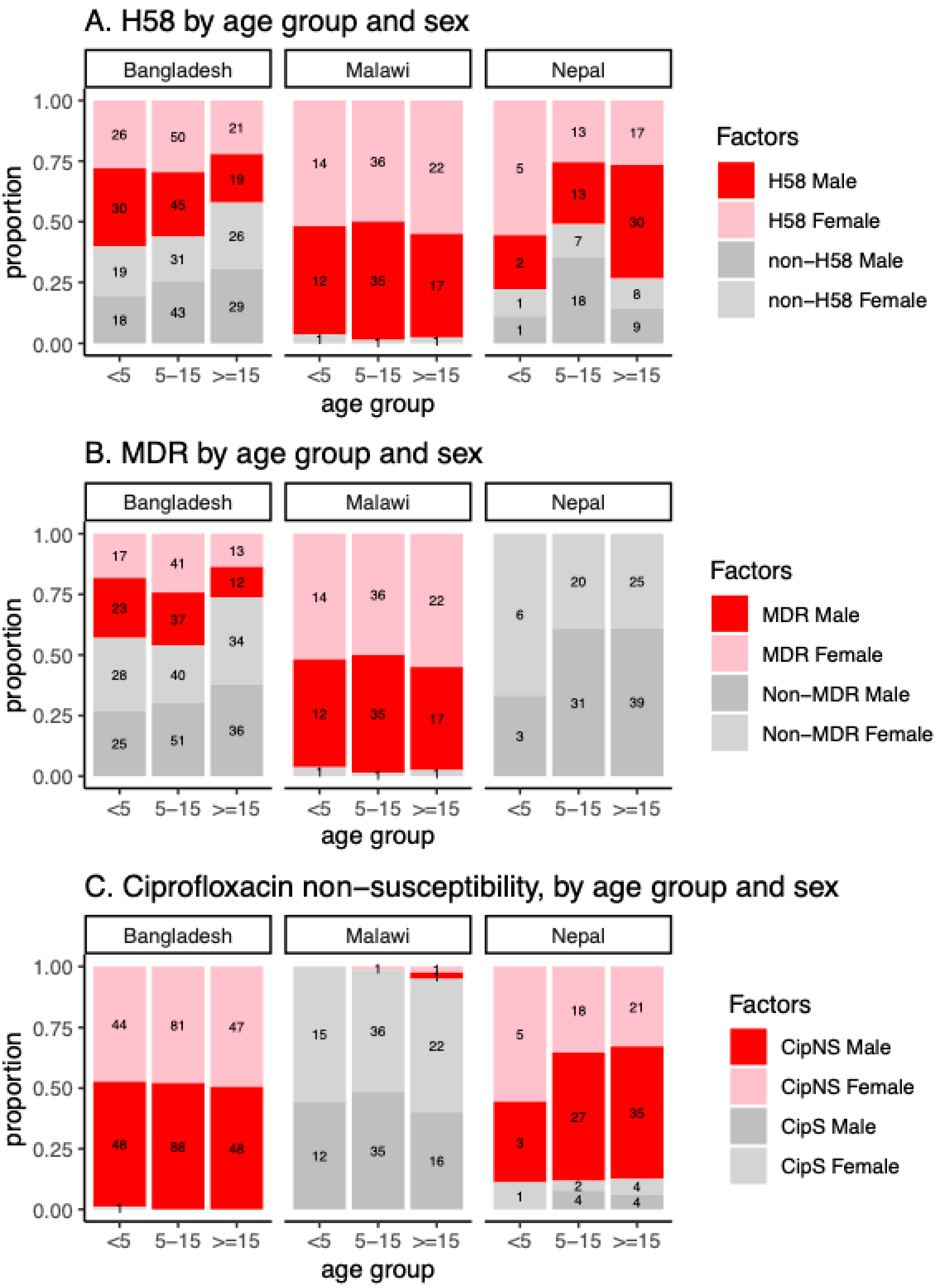

Figure S2. Breakdown of patient sex and Typhi features, within each age-group, at each site, for the main STRATAA dataset. (A) H58, genotype 4.3.1 and derived genotypes. **(B)** MDR, multi-drug resistant. **(C)** CipNS, ciprofloxacin non-susceptible; CipS, ciprofloxacin susceptible.

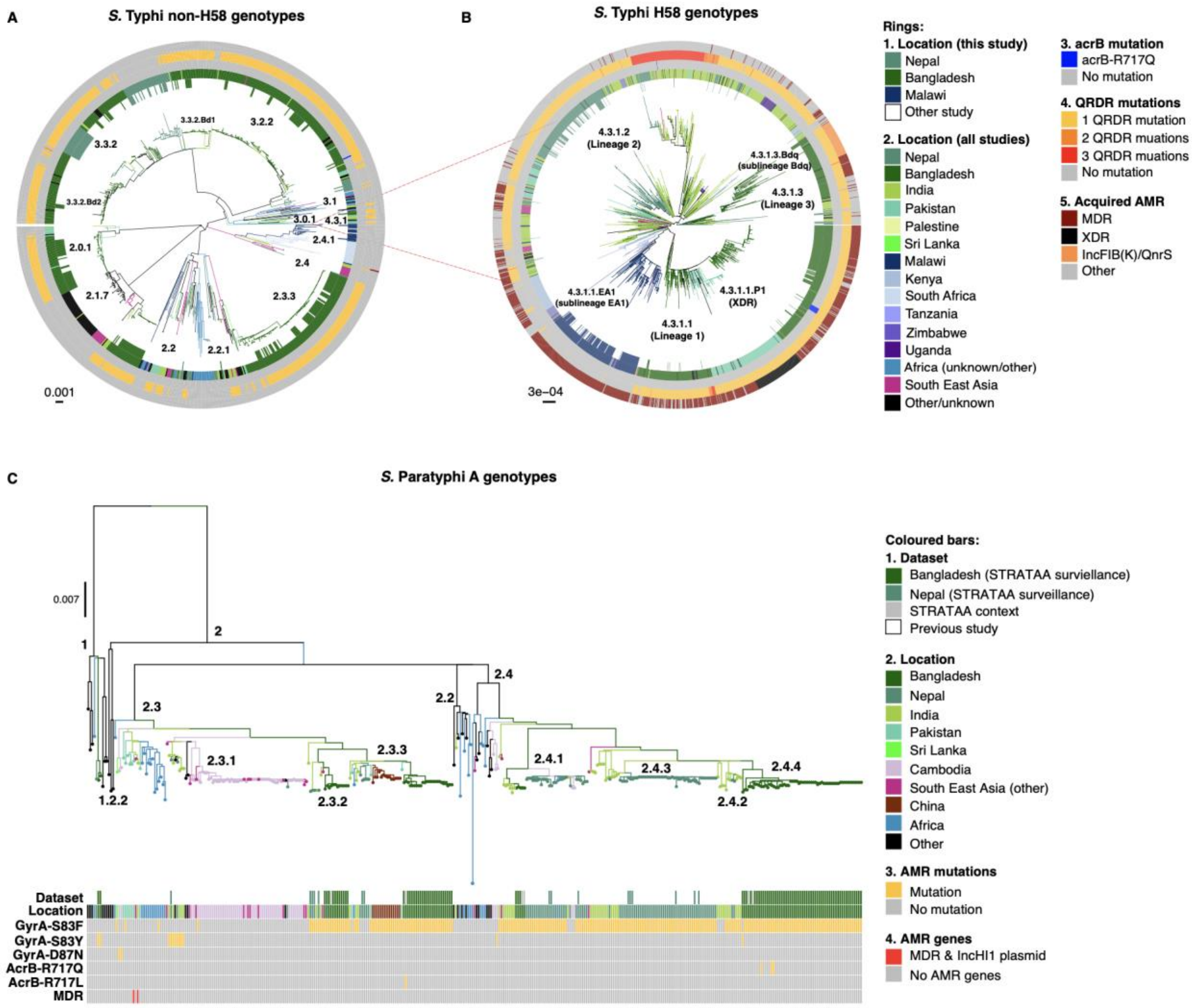

Figure S3. Phylogenetic trees of Typhi and Paratyphi A showing STRATAA isolate sequences in context with publicly available global genome data. (A) Non-H58 genomes (i.e., excluding 4.3.1 and derived genotypes). **(B)** Subtree of H58 (4.3.1 and derived) genotypes. A maximum-likelihood tree was inferred for n=3,835 Typhi sequences, including n=707 from this study plus global genomes, based on an alignment of genome-wide single nucleotide variants and outgroup rooted using Paratyphi A. The subtree of 4.3.1 genotypes (panel B) was pruned from the larger tree to aid visualisation. Genotypes are indicated with labels, and branch lengths indicate substitutions per variable site, as per inset scalebar. All branches and rings are coloured as per inset legend. Branch colours and first two rings indicate the geographic origin for the sequences; ring 1 highlights the n=707 sequences from this study. Other rings indicate antimicrobial resistance determinants as labelled. AcrB mutations are associated with azithromycin resistance. QRDR, quinolone resistance determining region. MDR, multidrug-resistant (MDR). XDR, extensively drug resistant. An interactive version of the full tree is available at https://microreact.org/project/wim5TssQ3AqSfgWXTSP2Bd.(C) Paratyphi A genomes. A maximum likelihood phylogeny was inferred in the same manner as for (A) from n=375 genomes and outgroup rooted using Typhi. Branch colours and the first two coloured bars indicate geographic origin of the sequences; bar 1 highlights the n=116 sequences from this study. Other coloured bars indicate antimicrobial resistance determinants as labelled. An interactive version of the full tree is available at: https://microreact.org/project/SgHbIR9cP.

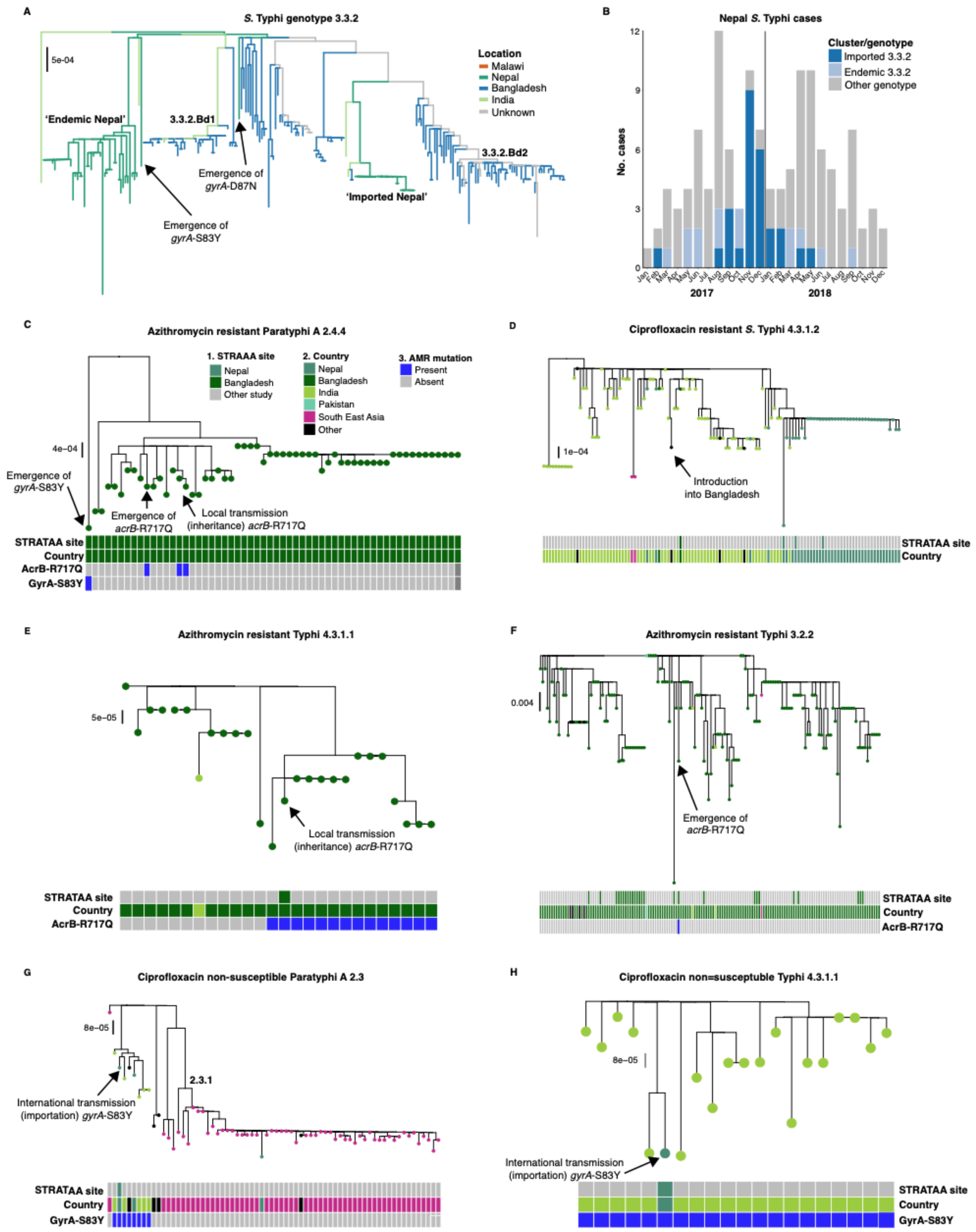

Figure S4. Detail of subpopulations discussed in text. Subtrees were extracted from the full tree of STRATAA plus global isolates (shown in Fig.S3) to highlight specific points discussed in the manuscript. **(A)** Typhi 3.3.2 tree, highlighting the two distinct clades identified as being either endemic or imported in Nepal. Arrows indicate the de novo emergence of ciprofloxacin non-susceptibility driven by non-synonymous mutations in gyrA (as labelled). **(B)** Epidemic curve of Typhi sequenced from STRATAA surveillance in Kathmandu, showing counts of imported and endemic genotype 3.3.2, defined using the tree in panel A. **(C)** Tree highlighting the phylogenetic positions of three *acrB* mutant (i.e. azithromycin-resistant) Paratyphi A 2.4.4 sequenced from STRATAA surveillance in Dhaka (labelled with arrows). The population is mainly *acrB*-wildtype; two STRATAA isolates appear to share an *acrB* mutation inherited from a common ancestor, consistent with local transmission of an azithromycin-resistant variant; the third *acrB* mutant is distantly related

from others and shows no evidence of transmission of a resistant ancestor. The emergence of the gyrA-S83Y mutation conferring ciprofloxacin non-susceptibility is also highlighted. **(D)** Tree highlighting the phylogenetic position of the single ciprofloxacin-resistant Typhi 4.3.1.2 sequenced from STRATAA surveillance in Dhaka (labelled with arrow), which suggests it was imported into Bangladesh (dark green) from India (light green) where it is believed to have emerged[13,43]. **(E)** Tree highlighting the phylogenetic position of the single *acrB* mutant of Typhi 4.3.1.1 sequenced from STRATAA surveillance in Dhaka (labelled with arrow), which suggests it results from local transmission of an *acrB* mutant (i.e. azithromycin-resistant) clade that has been detected in previous studies from Dhaka[30] (2013-2016). **(F)** Tree highlighting the phylogenetic position of the single *acrB* mutant (i.e. azithromycin-resistant) Typhi 3.2.2 sequenced from STRATAA surveillance in Dhaka (labelled with arrow); this suggests the mutation arose locally, in the background of a locally-circulating clade with wildtype *acrB. This* was categorised as a *de novo* resistance mutation. (G) Tree highlighting the phylogenetic position of the single gyrA-S83Y mutant (i.e. ciprofloxacin non-susceptible) Paratyphi A genotype 2.3 sequenced during STRATAA surveillance in Kathmandu (labelled with arrows). The position in the phylogeny suggests it was imported into Nepal (dark green) from India (light green). (H) Tree highlighting the phylogenetic position of the single gyrA-S83Y mutant (i.e. ciprofloxacin non-susceptible) Typhi genotype 4.3.1.1 sequenced during STRATAA surveillance in Kathmandu (labelled with arrows). The position in the phylogeny suggests it was imported into Nepal (dark green) from India (light green). For panels (**C-H**), branches are coloured by country of origin and coloured bars indicate samples from this study (STRATAA) by site, country of origin, and AcrB/GyrA status, as per the legend in panel (C).

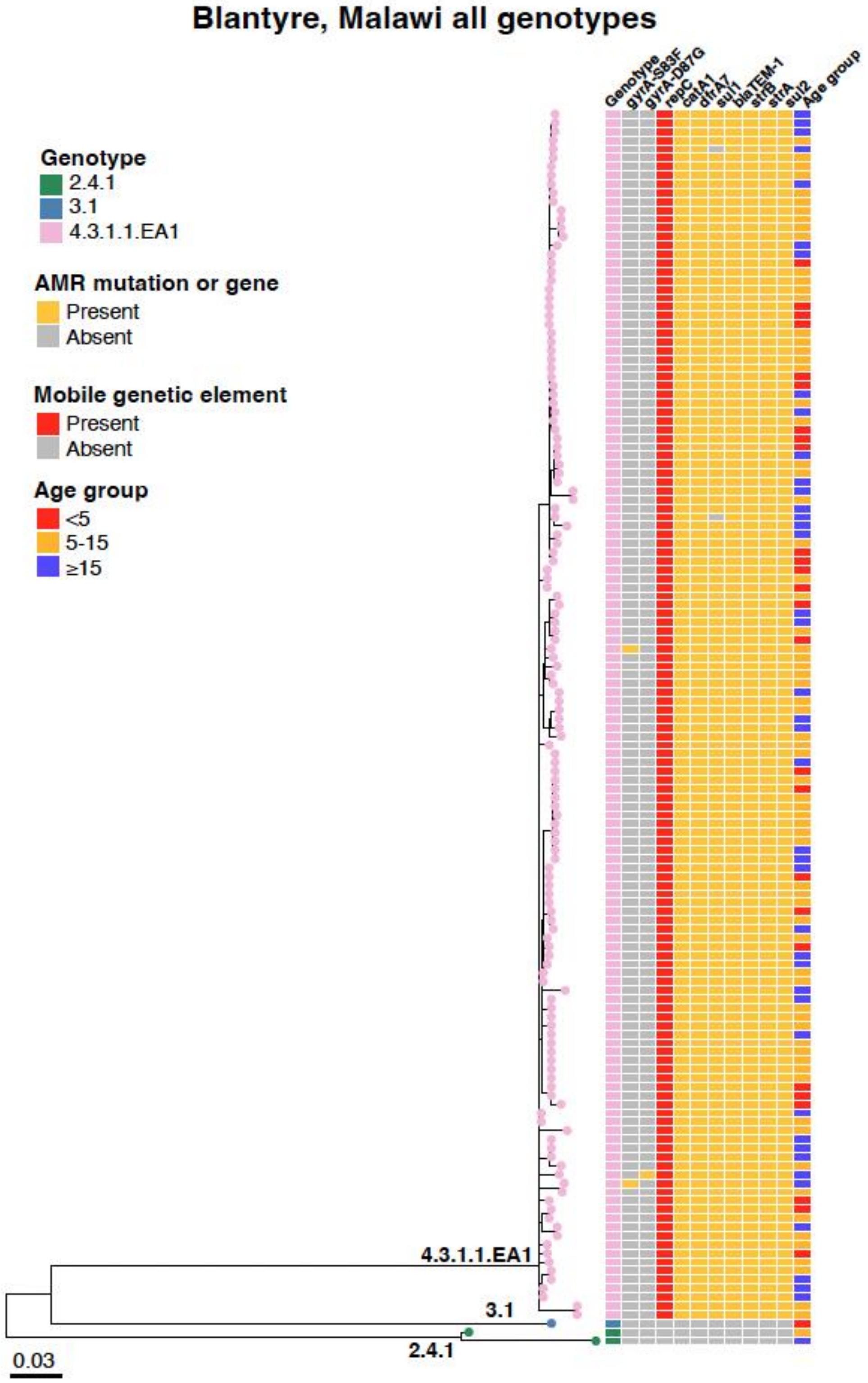

Figure S5. Phylogenetic tree of Typhi sequenced from STRATAA surveillance in Blantyre. Whole-genome maximum-likelihood phylogeny of n=141 Typhi sequences from Blantyre, Malawi (main 'STRATAA' dataset). Tip colours and first column of the heatmap indicate the genotype of the sequenced isolate (as per inset legend and branch labels). Second and third columns indicate presence of quinolone resistance-associated mutations in *gyrA*, remaining columns indicate presence of *repC* (a marker of the common multidrug resistant transposon), acquired genes associated with resistance to first-line drugs, and patient age group (coloured as per inset legends). Branch lengths indicate substitutions per variable site, as per the inset scalebar. Interactive phylogeny available at: https://microreact.org/project/beTX7esTof71d6J329iagM

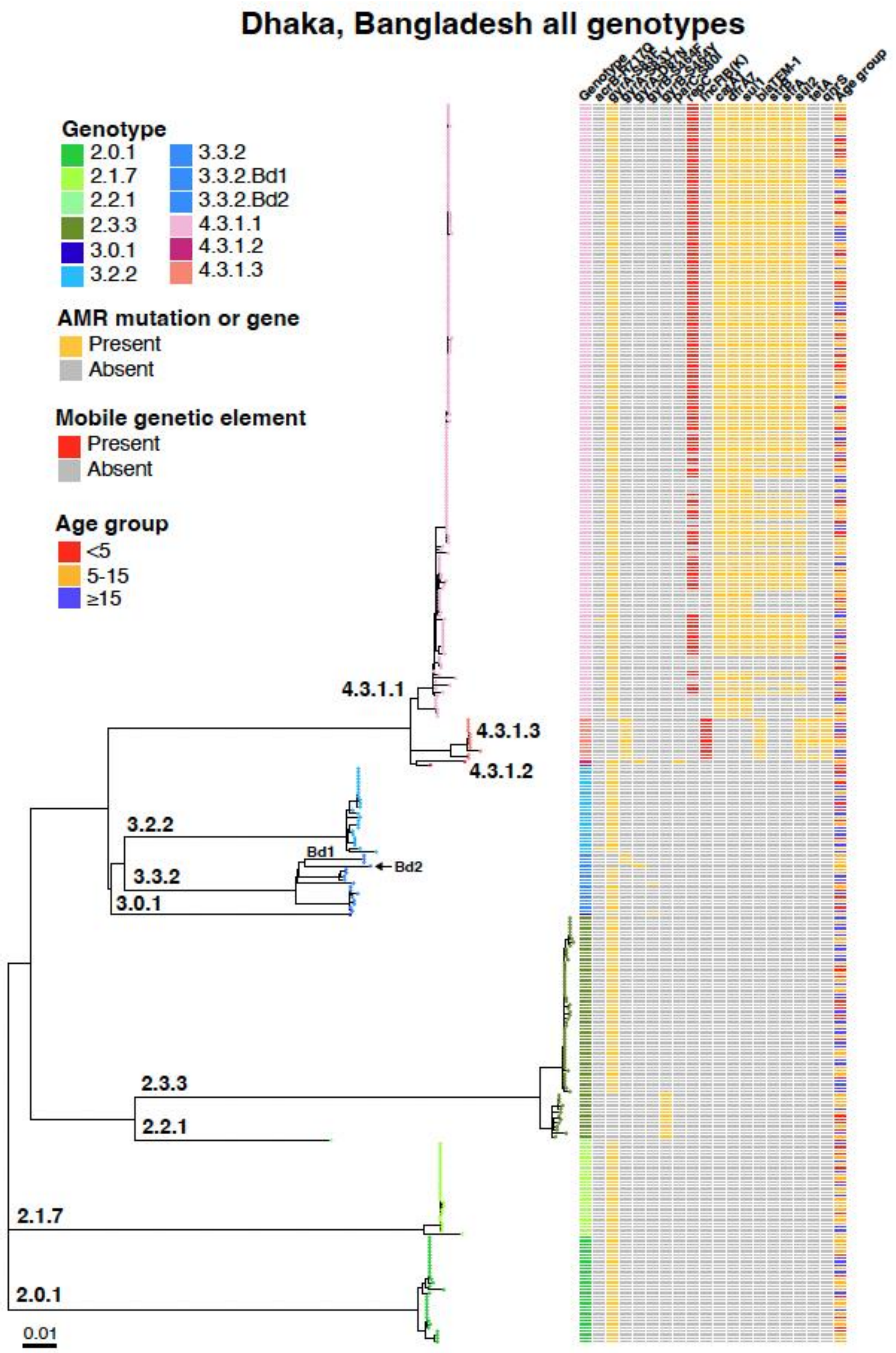

Figure S6. Phylogenetic tree of Typhi sequenced from STRATAA surveillance in Dhaka.
Whole-genome maximum-likelihood phylogeny of n=357 Typhi sequences from Dhaka, Bangladesh (main 'STRATAA' dataset). Tip colours and first column of heatmap indicate the genotype of the sequenced isolate (as per inset legend and branch labels). Second column indicates presence of azithromycin resistance-associated mutations in gene *acrB*. Subsequent columns indicate the presence of quinolone resistance-associated mutations in *gyrA* and *parC*; *repC* (a marker of the common multidrug resistant transposon); the IncFIBk plasmid replicon marker; acquired drug resistance genes, and patient age group (coloured as per inset legends). Branch lengths indicate substitutions per variable site, as per inset scalebar. Interactive phylogeny available at: https://microreact.org/project/eAY3YZmVd6tXbWKijzZ5Fc.

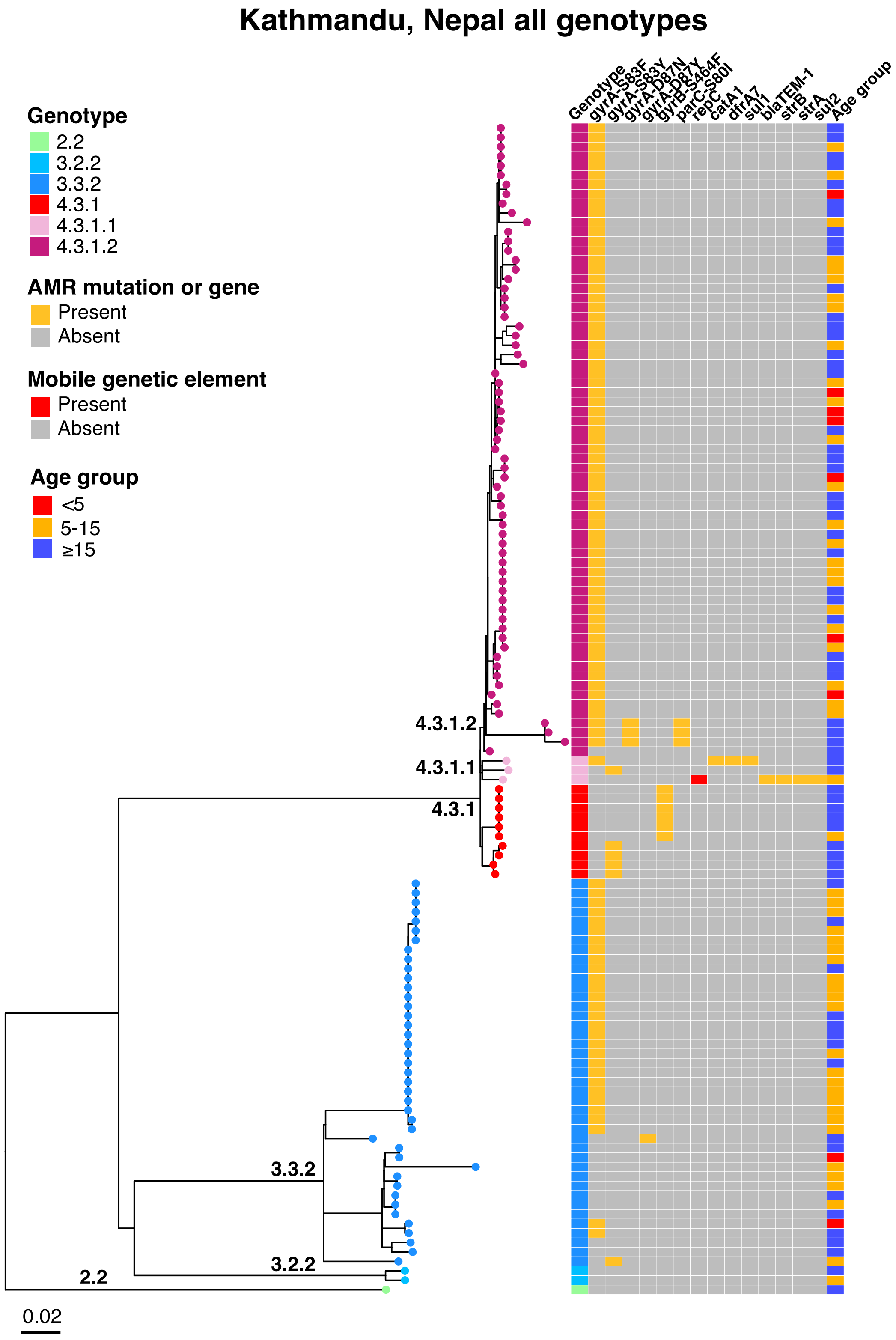

Figure S7. Phylogenetic tree of Typhi sequenced from STRATAA surveillance in Kathmandu. Whole-genome maximum-likelihood phylogeny of n=124 Typhi sequences from Kathmandu, Nepal (main 'STRATAA' dataset). Tip colours and first column of heatmap indicate the genotype of the sequenced isolate (as per inset legend and branch labels). 'Columns 2-7 indicate presence of quinolone resistance-associated mutations in *gyrA* and *parC*, remaining columns indicate presence of *repC* (a marker of the common multidrug resistant transposon), acquired genes associated with resistance to first-line drugs, and patient age group (coloured as per inset legends). Branch lengths indicate substitutions per variable site, as per inset scalebar. Interactive phylogeny available at: https://microreact.org/project/nJ42r2XHmd7gZCQ1eqQHGT.

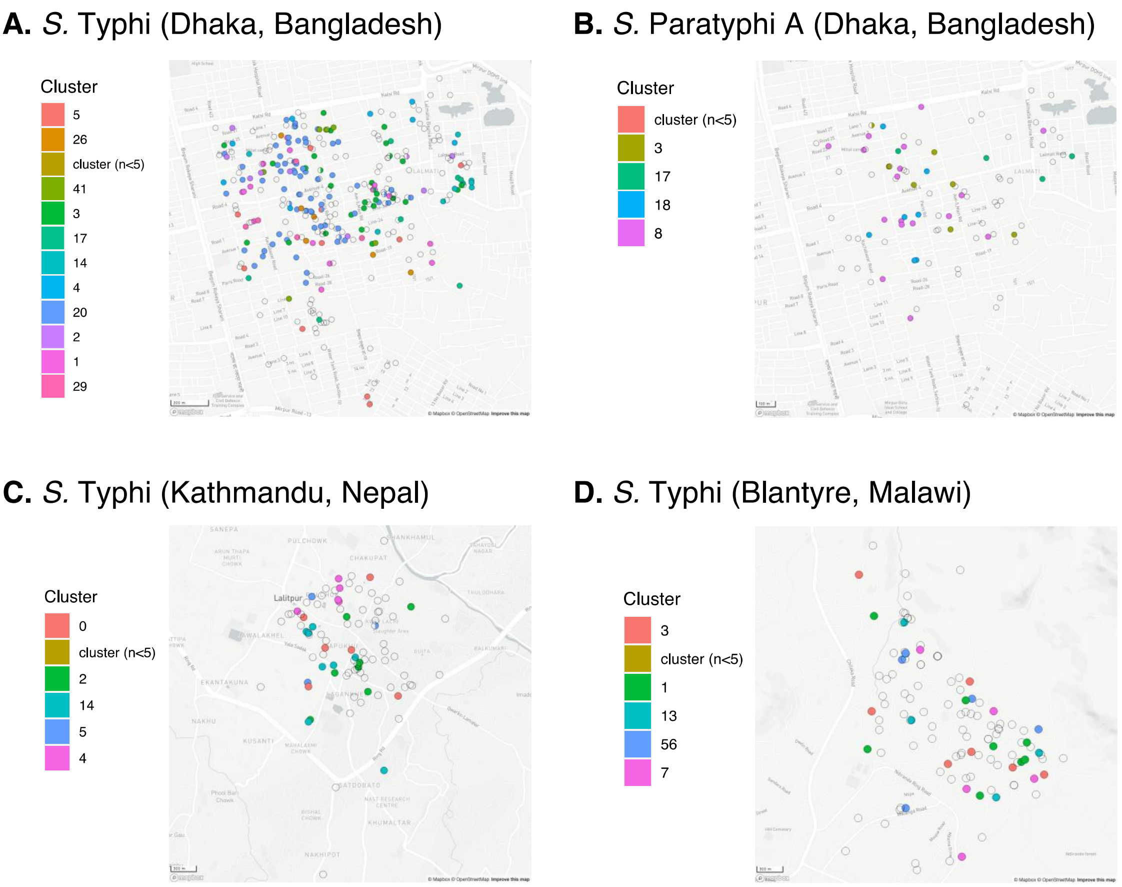

Figure S8. Spatial distribution of zero-SNV clusters from STRATAA surveillance. Each panel (A-D) shows the spatial distribution of clusters on a map. Filled points on the map each represent a sequenced case, coloured by the zero-SNV cluster as per inset legend in each panel (unfilled points indicate other cases, including unclustered cases or members of smaller clusters).

Country	Modelled Outcome	Predictor	p-value	OR (95% CI)
**Bangladesh**	**Serovar** Paratyphi A (n=95) vs Typhi (n=357)	Age group (≥5 and <15 years)	0·11	1·73 (0·89, 3·55)
		Age group (≥15 years)	$1.6x10^{-4}$	3·48 (1·79, 7·19)
		Sex (male)	0·20	1·36 (0·86, 2·17)
	**Typhi Genotype** Non-H58 Typhi (n=44) vs H58 Typhi (n=80)	Age group (≥5 and <15 years)	0·54	1·17 (0·71, 1·97)
		Age group (≥15 years)	0·01	2·07 (1·17, 3·71)
		Sex (male)	0·33	1·23 (0·81, 1·88)
	**Paratyphi A Genotype** 2.4.4 Paratyphi A (n=56) vs other Paratyphi A (n=39)	Age group (≥5 and <15 years)	0·04	0·23 (0·04, 0·91)
		Age group (≥15 years)	0·17	0·37 (0·07, 1·49)
		Sex (male)	0·84	1·09 (0·47, 2·54)
	**Typhi Multidrug Resistance** MDR Typhi (n=143) vs non-MDR Typhi (n=214)	Age group (≥5 and <15 years)	0·63	1·13 (0·68, 1·89)
		Age group (≥15 years)	0·02	0·48 (0·26, 0·87)
		Sex (male)	0·68	0·91 (0·60, 1·40)
**Nepal**	**Serovar** Paratyphi A (n=14) vs Typhi (n=124)	Age group (≥5 and <15 years)	0·90	1·12 (0·21, 11·4)
		Age group (≥15 years)	0·43	0·43 (0·07, 4·63)
		Sex (male)	0·75	1·20 (0·40, 3·92)
	**Typhi Genotype** Non-H58 Typhi (n=261) vs H58 Typhi (n=191)	Age group (≥5 and <15 years)	0·19	2·68 (0·63, 15·5)
		Age group (≥15 years)	0·97	1·03 (0·24, 5·96)
		Sex (male)	0·49	1·31 (0·61, 2·86)
	**Paratyphi A Genotype** 2.4.3 Paratyphi A (n=8) vs other Paratyphi A (n=3)	Age group (≥5 and <15 years)	0·31	0·18 (0·00, 4·73)
		Age group (≥15 years)	0·55	0·33 (0·00, 11·4)
		Sex (male)	0·97	0·97 (0·12, 8·60)
**Malawi**	**Typhi Genotype** Non-H58 Typhi (n=3) vs H58 Typhi (n=138)	Age group (≥5 and <15 years)	0·44	0·40 (0·03, 5·13)
		Age group (≥15 years)	0·72	0·65 (0·05, 8·48)
		Sex (male)	0.15	0·17 (0·00, 1.77)

Table S2. Logistic regression models for association of age and sex with pathogen features. Multivariable logistic regression models were fit separately for each country and outcome variable, with age group and sex as predictors. The total number (n) of individuals with each outcome variable, included in each model, is indicated. Age group was treated as a categorical variable: <5 years (reference category), ≥5 and <15 years, ≥15 years. OR, odds ratio; CI, confidence interval. As MDR pathogens were absent in Nepal and almost universal in Malawi, we did not assess association with age in these locations; similarly for ciprofloxacin susceptibility, which was almost invariant within sites, see Fig.S2c.

Country	Modelled Outcome	Predictor	p-value	OR (95% CI)
**Bangladesh**	**Severe disease** Yes (n=62) vs No (n=295)	Age group (≥5 and <15 years)	0·99	1·01 (0·51, 2·03)
		Age group (≥15 years)	0·33	1·44 (0·69, 3·06)
		Sex (male)	0·31	0·76 (0·44, 1·30)
		H58	0·21	0·60 (0·28, 1·35)
		MDR	0·55	0·78 (0·36, 1·78)
**Nepal**	**Severe disease** Yes (n=13) vs No (n=111)	Age group (≥5 and <15 years)	0·05	0·16 (0·03, 0·96)
		Age group (≥15 years)	0·14	0·30 (0·07, 1·51)
		Sex (male)	0·21	0·48 (0·14, 1·50)
		H58	0·90	1·08 (0·29, 3·58)
**Malawi**	**Severe disease** Yes (n=18) vs No (n=123)	Age group (≥5 and <15 years)	0·64	1·41 (0·36, 7·76)
		Age group (≥15 years)	0·17	2·68 (0·67, 15·0)
		Sex (male)	0·66	0·80 (0·28, 2·16)
		H58	0·27	3·65 (0·30, 32·1)
**All Sites**	**Severe disease** Yes (n=93) vs No (n=529)	Age group (≥5 and <15 years)	0·72	0·90 (0·50, 1·64)
		Age group (≥15 years)	0·31	1·37 (0·75, 2·57)
		Sex (male)	0·09	0·68 (0·43, 1·06)
		H58	0·29	0·69 (0·35, 1·38)
		MDR	0·65	0·84 (0·40, 1·80)
		Country (Malawi)	0·20	0·66 (0·34, 1·24)
		Country (Nepal)	0·04	0·47 (0·22, 0·98)

Table S3. Logistic regression models of severe Typhi infection.
Multivariable logistic regression models were fit separately for each country, and a combined model including all sites as indicated. The total number (n) of individuals with each outcome variable, included in each model, is indicated. OR, odds ratio; CI, confidence interval. Age group, sex and H58 genotype were predictors in all models. Age group was treated as a categorical variable: <5 years (reference category), ≥5 and <15 years, ≥15 years. Multidrug resistance (MDR) was included in the Bangladesh model as it varied across the population and with age, but was excluded from Nepal and Malawi models as it was invariant within those sites (near-universal in Malawi, absent in Nepal; see Fig.S2b). Quinolone resistance determining region (QRDR) mutation status was excluded from individual site models as it was invariant within sites; and from the all-sites model as it is confounded with country (see Fig.S2c).

Drug class	No. tested	**Resistant phenotype** N (% of total tested)	**Resistant genotype** N (% of total tested)	PPV	NPV
**Beta-lactamases**					
Ampicillin	575	287 (50%)	286 (50%)	99·7% [98·1, 100]	99·7% [98·1, 100]
Ceftriaxone	538	0	0	-	100% [99·3,100]
Cefixime	357	0	0	-	100% [99·0,100]
**Folate pathway inhibitors**					
Co-trimoxazole	552	304 (55%)	302 (55%)	98·7% [96·7,99·6]	99·2% [97·1,99·9]
**Carbapenems**					
Meropenem	354	0	0	-	100% [99·0,100]
**Macrolides**					
Azithromycin	469	13 (2·8%)	2 (0·4%)	50% [1·3,98·7]	97·4% [95·8,98·8]
**Chloramphenicol**					
Chloramphenicol	607	305 (50%)	301 (50%)	98·7% [96·7,99·6]	98·7% [96·6,99·6]
**Quinolones**					
Nalidixic Acid	444	437 (98%)	7 (1·6%)	100% [99·2,100]	98·4% [78·2,100]
**Fluoroquinolones**					
Ciprofloxacin	611	450 (74%)	444 (73%)	96·5% [94·4,98·0]	96·0% [91·6,98·5]

Table S4. Comparison of phenotypic and genotypic assessment of non-susceptibility to antimicrobials for Typhi. Drugs for which phenotype results were available for ≥20% of isolates are shown. Positive predictive value (PPV) and negative predictive value (NPV) of genetic determinants for prediction of resistant phenotypes are shown. Square brackets indicate 95% CI for PPV and NPV. Genetic determinants used as predictors of resistance are listed in Supplementary Methods.

Drug class	No. tested	Resistant phenotype N (% of total tested)	Resistant genotype N (% of total tested)	PPV	NPV
**Beta-lactamases**					
Ampicillin	104	0	0	-	100% [96·5, 100]
Ceftriaxone	99	0	0	-	100% [96·3, 100]
Cefixime	94	0	0	-	100% [96·2, 100]
**Folate pathway inhibitors**					
Co-trimoxazole	103	0	0	-	100% [96·5, 100]
**Carbapenems**					
Meropenem	93	0	0	-	100% [96·1, 100]
**Macrolides**					
Azithromycin	109	44 (40%)	3 (2·8%)	66·7% [9·4, 99·2]	60·4% [50·4, 69·8]
**Chloramphenicol**					
Chloramphenicol	109	0	0	-	100% [96·7, 100]
**Quinolones**					
Nalidixic Acid	109	109 (100%)	109 (100%)	100% [96·7, 100]	-
**Fluoroquinolones**					
Ciprofloxacin	109	108 (99%)	109 (100%)	99·1% [95·0, 100]	-

Table S5. Comparison of phenotypic and genotypic assessment of non-susceptibility to antimicrobials for Paratyphi A. Drugs for which phenotype results were available for ≥20% of isolates are shown. Positive predictive value (PPV) and negative predictive value (NPV) of genetic determinants for prediction of resistant phenotypes are shown. Square brackets indicate 95% CI for PPV and NPV. Genetic determinants used as predictors of resistance are listed in Supplementary Methods.

Supplementary References

1 Darton TC, Meiring JE, Tonks S, *et al.* The STRATAA study protocol: a programme to assess the burden of enteric fever in Bangladesh, Malawi and Nepal using prospective population census, passive surveillance, serological studies and healthcare utilisation surveys. *BMJ Open* 2017; **7**: e016283.

2 Meiring JE, Shakya M, Khanam F, *et al.* Burden of enteric fever at three urban sites in Africa and Asia: a multicentre population-based study. *Lancet Glob Health* 2021; **9**: e1688.

3 Patel JB, Cockerill FR, Bradford PA. Performance standards for antimicrobial susceptibility testing: twenty-fifth informational supplement. *Clinical and Laboratory Standards Institute* 2015.

4 Wong VK, Baker S, Pickard DJ, *et al.* Phylogeographical analysis of the dominant multidrug-resistant H58 clade of Salmonella Typhi identifies inter- and intracontinental transmission events. *Nat Genet* 2015; **47**: 632–9.

5 Parkhill J, Dougan G, James KD, *et al.* Complete genome sequence of a multiple drug resistant Salmonella enterica serovar Typhi CT18. *Nature* 2001; **413**: 848–52.

6 Holt KE, Thomson NR, Wain J, *et al.* Pseudogene accumulation in the evolutionary histories of Salmonella enterica serovars Paratyphi A and Typhi. *BMC Genomics* 2009; **10**: 36.

7 Langmead B, Salzberg SL. Fast gapped-read alignment with Bowtie 2. *Nat Meth* 2012; **9**: 357–9.

8 Li H, Durbin R. Fast and accurate long-read alignment with Burrows–Wheeler transform. *Bioinformatics* 2010; **26**: 589–95.

9 Britto CD, Dyson ZA, Duchene S, *et al.* Laboratory and molecular surveillance of paediatric typhoidal Salmonella in Nepal: Antimicrobial resistance and implications for vaccine policy. *PLoS Negl Trop Dis* 2018; **12**: e0006408.

10 Dyson ZA, Holt KE. Five Years of GenoTyphi: Updates to the Global Salmonella Typhi Genotyping Framework. *J Infect Dis* 2021; **224**: S775–80.

11 Wong VK, Baker S, Connor TR, *et al.* An extended genotyping framework for Salmonella enterica serovar Typhi, the cause of human typhoid. *Nat Commun* 2016; **7**: 12827.

12 Tanmoy AM, Hooda Y, Sajib MSI, *et al.* Paratype: a genotyping tool for Salmonella Paratyphi A reveals its global genomic diversity. *Nat Commun* 2022; **13**: 7912.

13 Britto CD, Dyson ZA, Mathias S, *et al.* Persistent circulation of a fluoroquinolone-resistant Salmonella enterica Typhi clone in the Indian subcontinent. *J Antimicrob Chemother* 2020; **75**: 337–41.

14 Tanmoy AM, Westeel E, Bruyne KD, *et al.* Salmonella enterica Serovar Typhi in Bangladesh: Exploration of Genomic Diversity and Antimicrobial Resistance. *mBio* 2018; **9**: e02112-18.

15 Dyson ZA, Thanh DP, Bodhidatta L, *et al.* Whole Genome Sequence Analysis of Salmonella Typhi Isolated in Thailand before and after the Introduction of a National Immunization Program. *PLoS Negl Trop Dis* 2017; **11**: e0005274.

16 Klemm EJ, Shakoor S, Page AJ, *et al.* Emergence of an Extensively Drug-Resistant Salmonella enterica Serovar Typhi Clone Harboring a Promiscuous Plasmid Encoding Resistance to Fluoroquinolones and Third-Generation Cephalosporins. *mBio* 2018; **9**. DOI:10.1128/mbio.00105-18.

17 Matono T, Morita M, Yahara K, *et al.* Emergence of Resistance Mutations in Salmonella enterica Serovar Typhi Against Fluoroquinolones. *Open Forum Infect Dis* 2017; **4**: ofx230.

18 Pragasam AK, Pickard D, Wong V, *et al.* Phylogenetic Analysis Indicates a Longer Term Presence of the Globally Distributed H58 Haplotype of Salmonella Typhi in Southern India. *Clin Infect Dis* 2020; **71**: 1856–63.

19 Rahman SIA, Dyson ZA, Klemm EJ, *et al.* Population structure and antimicrobial resistance patterns of Salmonella Typhi isolates in urban Dhaka, Bangladesh from 2004 to 2016. *PLoS Negl Trop Dis* 2020; **14**: e0008036.

20 Rasheed F, Saeed M, Alikhan N-F, *et al.* Emergence of Resistance to Fluoroquinolones and Third-Generation Cephalosporins in Salmonella Typhi in Lahore, Pakistan. *Microorganisms* 2020; **8**: 1336.

21 Thanh DP, Karkey A, Dongol S, *et al.* A novel ciprofloxacin-resistant subclade of H58 Salmonella Typhi is associated with fluoroquinolone treatment failure. *eLife* 2016; **5**: e14003.

22 International Typhoid Consortium, Wong VK, Holt KE, *et al.* Molecular Surveillance Identifies Multiple Transmissions of Typhoid in West Africa. *PLoS Negl Trop Dis* 2016; **10**: e0004781.

23 Holt KE, Parkhill J, Mazzoni CJ, *et al.* High-throughput sequencing provides insights into genome variation and evolution in Salmonella Typhi. *Nat Genet* 2008; **40**: 987–93.

24 Ingle DJ, Nair S, Hartman H, *et al.* Informal genomic surveillance of regional distribution of Salmonella Typhi genotypes and antimicrobial resistance via returning travellers. *PLoS Negl Trop Dis* 2019; **13**: e0007620.

25 Croucher NJ, Page AJ, Connor TR, *et al.* Rapid phylogenetic analysis of large samples of recombinant bacterial whole genome sequences using Gubbins. *Nucleic Acids Research* 2015; **43**: e15–e15.

26 Stamatakis A. RAxML version 8: a tool for phylogenetic analysis and post-analysis of large phylogenies. *Bioinformatics* 2014; **30**: 1312–3.

27 Pattengale ND, Alipour M, Bininda-Emonds ORP, Moret BME, Stamatakis A. How Many Bootstrap Replicates Are Necessary? *J Comput Biol* 2010; **17**: 337–54.

28 Argimon S, Abudahab K, Goater RJE, *et al.* Microreact: visualizing and sharing data for genomic epidemiology and phylogeography. *Microbial Genomics* 2016; **2**: e000093.

29 Yu G, Smith DK, Zhu H, Guan Y, Lam TT-Y. ggtree: an rpackage for visualization and annotation of phylogenetic trees with their covariates and other associated data. *Methods Ecol Evol* 2016; **8**: 28–36.

30 Hooda Y, Sajib MSI, Rahman H, *et al.* Molecular mechanism of azithromycin resistance among typhoidal Salmonella strains in Bangladesh identified through passive pediatric surveillance. *PLoS Negl Trop Dis* 2019; **13**: e0007868.

31 Argimon S, Yeats CA, Goater RJ, *et al.* A global resource for genomic predictions of antimicrobial resistance and surveillance of Salmonella Typhi at pathogenwatch. *Nat Commun* 2021; **12**: 2879–12.

32 Inouye M, Dashnow H, Raven L-A, *et al.* SRST2: Rapid genomic surveillance for public health and hospital microbiology labs. *Genome Med* 2014; **6**: 90.

33 Gupta SK, Padmanabhan BR, Diene SM, *et al.* ARG-ANNOT, a new bioinformatic tool to discover antibiotic resistance genes in bacterial genomes. *Antimicrob Agents Chemother* 2014; **58**: 212–20.

34 Carattoli A, Zankari E, García-Fernández A, *et al.* In silico detection and typing of plasmids using PlasmidFinder and plasmid multilocus sequence typing. *Antimicrob Agents Chemother* 2014; **58**: 3895–903.

35 Chattaway MA, Gentle A, Nair S, *et al.* Phylogenomics and antimicrobial resistance of Salmonella Typhi and Paratyphi A, B and C in England, 2016–2019. *Microb Genom* 2021; **7**: 000633.

36 Stevenson M, Stevenson MM, BiasedUrn I. Package 'epiR.' *Tools for the analysis of epidemiological data R package version 09--62* 2015.

37 Wilm A, Aw PPK, Bertrand D, *et al.* LoFreq: a sequence-quality aware, ultra-sensitive variant caller for uncovering cell-population heterogeneity from high-throughput sequencing datasets. *Nucleic Acids Research* 2012; **40**: 11189–201.

38 Knaus BJ, Grünwald NJ. vcfr: a package to manipulate and visualize variant call format data in R. *Molecular Ecology Resources* 2017; **17**: 44–53.

39 Jaillard M, Lima L, Tournoud M, *et al.* A fast and agnostic method for bacterial genome-wide association studies: Bridging the gap between k-mers and genetic events. *PLoS Genet* 2018; **14**: e1007758.

40 Wick RR, Judd LM, Gorrie CL, Holt KE. Unicycler: Resolving bacterial genome assemblies from short and long sequencing reads. *PLoS Comput Biol* 2017; **13**: e1005595.

41 Schliep KP. phangorn: phylogenetic analysis in R. *Bioinformatics* 2011; **27**: 592–3.

42 Heinze G, Ploner M, Dunkler D, Southworth H, Heinze MG. Package 'logistf.' .

43 Silva KE da, Tanmoy AM, Pragasam AK, *et al.* The international and intercontinental spread and expansion of antimicrobial-resistant Salmonella Typhi: a genomic epidemiology study. *Lancet Microbe* 2022; **3**: e567–77.
